# Expression and significance of m6A-RNA-methylation in oral cancer and precancerous lesion

**DOI:** 10.3389/fonc.2023.1013054

**Published:** 2023-01-30

**Authors:** Zhiming Qin, Jiaying Bai, Huiying He, Binbin Li

**Affiliations:** ^1^ Department of Oral Pathology, Peking University School and Hospital of Stomatology & National Center of Stomatology & National Clinical Research Center for Oral Diseases & National Engineering Research Center of Oral Biomaterials and Digital Medical Devices, Beijing, China; ^2^ Research Unit of Precision Pathologic Diagnosis in Tumors of the Oral and Maxillofacial Regions, Chinese Academy of Medical Sciences (2019RU034), Beijing, China; ^3^ Department of Pathology, School of Basic Medical Sciences, Third Hospital, Peking University Health Science Center, Beijing, China

**Keywords:** oral squamous cell carcinoma, oral epithelial dysplasia, N6-methyladenosine, IGF2BP2, IGF2BP3

## Abstract

**Background:**

Oral potentially malignant disorders (OPMDs) and oral squamous cell carcinoma (OSCC) are a series of related pathologic and molecular events involving simple epithelial hyperplasia, mild to severe dysplasia and canceration. N6-methyladenosine RNA methylation, as the most common modification of both coding mRNA and non-coding ncRNA in eukaryotes, participates in the regulation of the occurrence and development of various malignant tumors in human. However, its role in oral epithelial dysplasia (OED) and OSCC remain unclear.

**Materials and methods:**

In this study, multiple public databases were used for bioinformatics analysis of 23 common m6A methylation regulators in head and neck squamous cell carcinoma (HNSCC). Protein expressions of IGF2BP2 and IGF2BP3 were verified accordingly in clinical cohort samples of OED and OSCC.

**Results:**

Patients with high expression of FTO、HNRNPC、HNRNPA2B1、LRPPRC、IGF2BP1、IGF2BP2、IGF2BP3 had a poor prognosis. IGF2BP2 had a relatively high mutation rate in HNSCC, and its expression was significantly positively correlated with tumor purity, and significantly negatively correlated with the infiltration level of B cells and CD8+T cells. The expression of IGF2BP3 was significantly positively correlated with tumor purity and CD4+T cells. Immunohistochemistrically, the expression of IGF2BP2 and IGF2BP3 in oral simple epithelial hyperplasia, OED and OSCC increased gradually. Both were strongly expressed in OSCC.

**Conclusion:**

IGF2BP2 and IGF2BP3 were the potential biological prognostic indicators of OED and OSCC.

## Introduction

Oral squamous cell carcinoma (OSCC) is the most prevalent malignancy in oral and maxillofacial region, with approximately 350,000 newly diagnosed cases annually (1). A considerable number of patients with OSCC develop from oral epithelial dysplasia (OED). It is generally believed that the higher the degree of epithelial dysplasia, the higher the risk of malignant transformation. However, the pathological judgment is subjective to some extent, which may lead to discrepancy between different pathologists. In some cases, the pathologic predictions had no significant relation with clinical prognosis. Therefore, it is needed to discover more molecular markers for accurate assessment of cancer risk and further determine the specific biomarkers of OED and OSCC.

N6-methyladenosine (m6A), the methylation modification at the sixth N atom of adenine, is the most common post-transcriptional modification on mRNA, mediating >60% RNA methylation (2). Abnormal m6A methylation levels play an essential role in the progression of various cancers (3, 4). M6A methylation is dynamic and reversible in cells, and the levels of m6A methylation in tumors mainly depend on the expression of m6A methylation regulators. Regulators that interact with m6A methylation are mainly divided into three categories: Writers (methyltransferases), Erasers (demethylases), and Readers (reading proteins). Recently, new evidence has showed that m6A modification is associated with tumor proliferation, glycolysis, apoptosis, and metastasis. m6A modification could play either an oncogenetic or tumor-suppressive role in malignant tumors (5, 6). For example, METTL3, as one of the m6A-RNA-methylation regulators, is upregulated in OSCC tissues, and high levels of METTL3 expression in tumor tissues predict poor patient survival. METTL3 promotes the proliferation, invasion, and migration of OSCC cells *in vitro*, while METTL3-knockout inhibits tumor growth *in vivo*. METTL3 promotes the stabilization of c-Myc through YTHDF1-mediated m6A modification, leading to the occurrence of OSCC (7). In addition, OSCC patients with high FTO expression had larger tumor volume, higher tumor node metastasis (TNM) stage, worse differentiation, and shorter survival time. Stable knockout of FTO inhibited OSCC cell viability, colony formation, and tumor growth (8).

Thus, we utilized public databases to analyze the expression of 23 m6A methylation regulators and their relationships with clinicopathological characteristics, and to predict their potential functions. Further, clinical cohort cases with epithelial hyperplasia/dysplasia and OSCC were collected to verify the protein expression of Insulin-Like Growth Factor 2 mNA Binding Protein 2 (IGF2BP2) and Insulin-Like Growth Factor 2 mNA Binding Protein 3 (IGF2BP3), and their clinical associations in OSCC and OED cases.

## Materials and methods

### Oncomine analysis

Oncomine gene expression array datasets (https://www.oncomine.org/resource/login.html) were used to analyze the transcription levels of m6A regulators in cancers. The cutoffs of *P*-value and fold change were defined as 0.01 and 2 by Student’s t-test, respectively.

### Gene expression profiling interactive analysis dataset

GEPIA is a newly developed interactive web server for analyzing the RNA sequencing (RNA-Seq) expression data of 9,736 tumors and 8,587 normal samples from the Cancer Genome Atlas and the Genotype-Tissue Expression projects, using a standard processing pipeline. GEPIA provides customizable functions such as tumor/normal differential expression analysis, profiling according to cancer types or pathological stages, patient survival analysis, similar gene detection, correlation analysis, and dimensionality reduction analysis (9).

### The Kaplan-Meier Plotter

Kaplan-Meier Plotter (www.kmplot.com) was used to analyze the overall survival (OS), first-progression survival (FP), and post-progression survival (PPS) of patients with head and neck squamous cell carcinoma (HNSCC), which contained gene expression data and survival information of HNSCC patients (10). Cases were divided into two groups by median expression (high vs low expression) and assessed by a Kaplan-Meier survival plot, with the hazard ratio (HR) with 95% confidence intervals (CIs) and log-rank p-value. Only the JetSet best probe set of m6A regulators was chosen to obtain Kaplan-Meier plots, in which the number-at-risk was indicated below the main plot.

### The cBioPortal

The Cancer Genome Atlas have sequencing and pathological data of 30 different cancers. The HNSCC dataset, including data from 1,107 cases with pathology reports, was selected for further analyses of M6A regulators using cBioPortal (11). The genomic profiles included mutations, putative copy number alterations (CNAs) from genomic identification of significant targets in cancer (GISTIC), mRNA expression Z scores (RNA-seq v.2 RSEM), and protein expression Z scores (reverse phase protein array).

### The TIMER database analysis

The Timer database is a publicly available data platform for the systematic analysis of tumor immune infiltration (12). We used GENE modules to explore the correlation between the expression levels of m6A methylation regulators and immune cell infiltration in patients with HNSCC, including CD4+ T cells, CD8+ T cells, B cells, neutrophils, dendritic cells and macrophages.

### Patient sections

The study samples were obtained from Peking University School and Hospital of Stomatology (Beijing, China) from January 2000 to June 2021. Inclusion criteria were as follows: the initial diagnosis was epithelial hyperplasia or epithelial dysplasia; there were complete medical records and clinical data; and there was no history of oral mucosal epithelial lesions or OSCC. Exclusion criteria were as follows: no pathological biopsy was performed; cancerous change was diagnosed by pathological biopsy at the first visit; and there was a history of radiotherapy and chemotherapy in the past. All study samples were further confirmed by two pathologists according to World Health Organization criteria (13). This study was in compliance with the Declaration of Helsinki. Data were collected after approval of the Biomedical Ethics Committee of Peking University School and Hospital of Stomatology (No. PKUSSIRB-201948111).

### Immunohistochemistry

Formalin-fixed and paraffin-embedded (FFPE) tissues were chosen from above cases. Briefly, 3-mm sections were incubated with commercial rabbit poly-clonal antibodies against IGF2BP2 and IGF2BP3 (Abcam) at 1/200 dilution overnight at 4°C. Then, the sections were conjugated with horse-radish peroxidase (HRP) antibody (1:500 dilution; Gene Tech) at room temperature for 2 h, then covered by 3, 3-diaminobenzidine (DAB), and slides were mounted with mounting medium.

### Statistical analysis

All analyses were performed with the SPSS 24.0 software (SPSS Inc., USA) and the data from these independent experiments were presented as mean ± SD. If the multi-sample comparisons were independent of each other and conform to the normal distribution and homogeneity of variance, a one-way analysis of variance would be applied. Differences between groups were compared using the least significant difference (LSD) test. If the heterogeneity between samples didn’t conform to the normal distribution, the Nonparametric tests Mann-Whitney test, or Kruskal-Wallis H test would be used. Enumeration data were analyzed using Fisher’s exact test of χ2 test. Correlation analysis was performed using Spearman correlation analysis. The test level α= 0.05 and P<0.05 was considered statistically significant.

## Results

### Baseline characteristics of the patient sections

A total of 109 patients with OSCC or OED were enrolled. In this cohort, 28 patients relapsed after the initial biopsy and became cancer, and 81 patients underwent no recurrence within 5 years. Among them, 48 sections were assigned to the mild epithelial dysplasia group, 71 sections were assigned to the moderate epithelial dysplasia group, 57 sections were assigned to the severe epithelial dysplasia group, 63 sections were assigned to the OSCC group, and 37 sections were assigned to the epithelial hyperplasia group.

At the same time, 195 biopsy sections were collected from above 28 patients with malignant transformation, and only one section was selected for each biopsy, which were recorded as the progression group; 81 patients were pathologically diagnosed as epithelial dysplasia at the first visit, and there was no recurrence or malignant transformation during the follow-up period of at least 5 years, which were collected as the stable group (**Tables 1**, **2**).

**Table 1:** Baseline characteristics of the patients in advanced group.

Characteristic	Epithelial hyperplasia		Mild dysplasia		Moderate dysplasia		Severe dysplasia	
	Number %		Number %		Number %		Number %	
Sex								
** Female**	6	85.71	4	80.00	8	61.54	3	100.00
** Male**	1	14.29	1	20.00	5	38.46	0	0.00
Age(years)								
** ≤40**	0	0.00	1	20.00	1	7.69	0	0.00
** 40-60**	5	71.43	1	20.00	7	53.85	0	0.00
** ≥60**	2	28.57	3	60.00	5	38.46	3	100.00
Sites								
** Tongue**	2	28.57	3	60.00	6	46.15	1	33.33
** Buccal**	2	28.57	1	20.00	1	7.69	1	33.33
** Gingiva**	3	42.86	1	20.00	3	23.08	1	33.33
** Other**	0	0.00	0	0.00	3	23.08	0	0.00
Tobacco smoking								
** Yes**	0	0.00	0	0.00	2	15.38	0	0.00
** No**	7	100.00	5	100.00	11	84.62	3	100.00
Alcohol drinking								
** Yes**	0	0.00	0	0.00	1	7.69	0	0.00
** No**	7	100.00	5	100.00	12	92.31	3	100.00
Prognosis(relapse)								
** Original sites**	3	42.86	3	60.00	8	61.54	1	33.33
** Multiple sites**	4	57.14	2	40.00	5	38.46	2	66.67

**Table 2:** Baseline characteristics of the patients in stable group.

Characteristic	Epithelial hyperplasia		Mild dysplasia		Moderate dysplasia		Severe dysplasia	
	Number %		Number %		Number %		Number %	
Sex								
** Female**	10	47.62	8	40.00	6	31.58	11	52.38
** Male**	11	52.38	12	60.00	13	68.42	10	47.62
Age(years)								
** ≤40**	7	33.33	3	15.00	1	8.86	1	4.76
** 40-60**	11	52.38	11	55.00	11	57.89	9	42.86
** ≥60**	3	14.29	6	30.00	7	36.84	11	52.38
Sites								
** Tongue**	12	57.14	6	30.00	12	63.16	13	61.90
** Buccal**	6	28.57	10	50.00	3	15.79	0	0.00
** Gingiva**	3	14.29	3	15.00	2	10.53	3	14.29
** Other**	0	0.00	1	5.00	2	10.53	4	19.05
Tobacco smoking								
** Yes**	6	28.57	10	50.00	3	15.79	7	33.33
** No**	15	71.43	10	50.00	16	84.21		66.67
Alcohol drinking								
** Yes**	6	28.57	5	25.00	3	15.79	8	38.10
** No**	15	71.43	15	75.00	16	84.21	13	61.90

### Bioinformatic analysis of m6A methylation regulators in HNSCC

**Figure 1:**
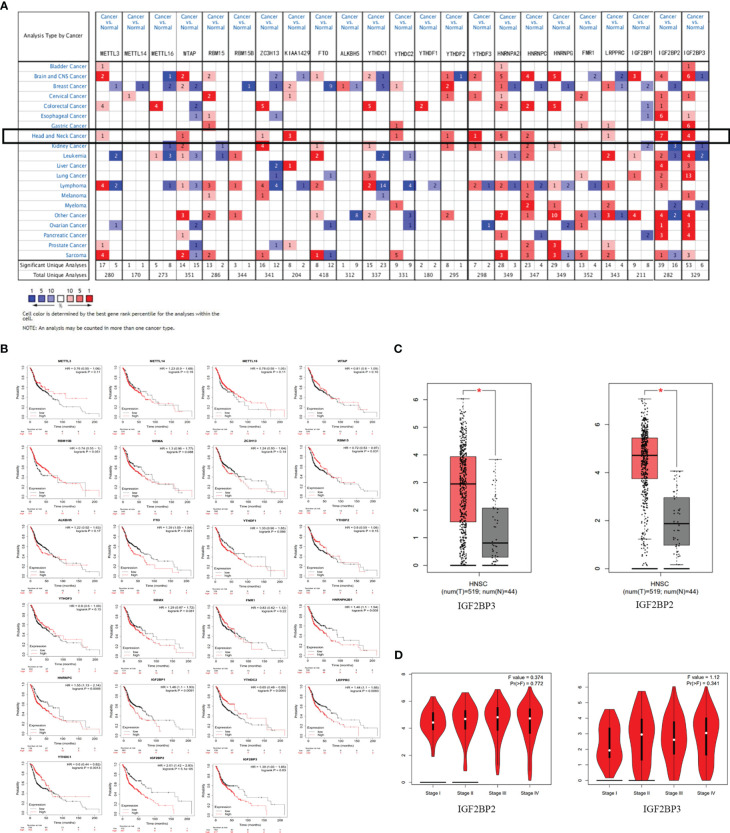
m6A and its regulatory genes abnormally expressed in head and neck squamous cell carcinoma (HNSCC). **(A)** An integrated analysis of 23 m6A methylation regulators by searching the Oncomine database. **(B)** The correlation between m6A methylation regulators and HNSCC prognosis supported by the data from Kaplan-Meier Plotter database. **(C)** The expressions of IGF2BP2 and IGF2BP3 were upregulated in HNSCC by searching the GEPIA database. **(D)** No significant difference between IGF2BP2 and IGF2BP3 in different stages of HNSCC (*P*>0.05) from the data of GEPIA database.

A lot of evidence showed that m6A RNA methylation promotes tumor initiation and progression (14). However, there are few studies to explore the role of m6A methylation in OSCC and OED. To study the expression correlation of m6A methylation regulators in OSCC and OED, we reviewed relevant literature and selected a total of 23 m6A methylation regulators. We preliminarily analyzed the expression of these regulators in different cancer types through the Oncomine database compared with normal control. **Figure 1A** demonstrated that the m6A level upregulated in OSCC and OED tissues compared with normal control. It was found that m6A methylation regulators were expressed to different degrees in various cancer types, and the expressions of METTL3, WTAP, ZC3H13, KIAA1429, YTHDC, YTHDF2, YTHDF3, HNRNPA2B, HNRNPC, LRPPRC, IGF2BP2 and IGF2BP3 in HNSCC were higher, among which KIAA1429, YTHDF3, IGF2BP2, and IGF2BP3 had significant differences in the corresponding studies (**Supplementary Material**)

**Figure 1B** showed that the expression levels of RBM15, FTO, YTHDC1, YTHDC2, HNRNPC, HNRNPA2B1, LRPPRC, IGF2BP1, IGF2BP2, IGF2BP3 were significantly correlated with the overall survival of patients with HNSCC (P<0.05) through Kaplan-Meier curve and log-rank test analysis. Among them, the higher the expression of RBM15, YTHDC1, and YTHDC2, the better the prognosis of the patient; while the overall survival time of patients with high expressions of FTO, HNRNPC, HNRNPA2B1, LRPPRC, IGF2BP1, IGF2BP2, and IGF2BP3 were significantly shorter than those of patients with low expressions.

**Figure 2:**
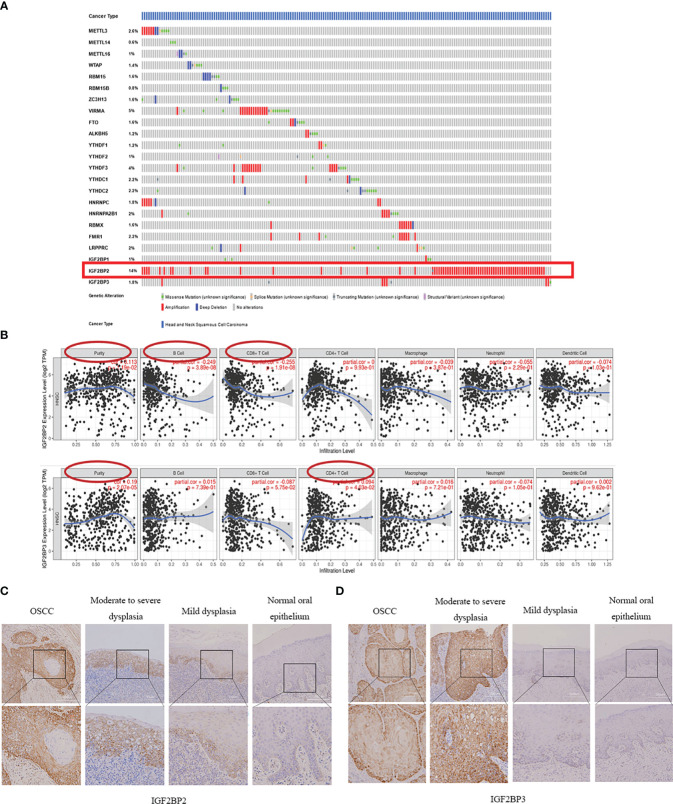
Mutation frequency of m6A methylation regulators in HNSCC and their association with IGF2BP2 and IGF2BP3. **(A)** The mutation frequency of m6A methylation regulators in HNSCC (The cBioPortal database). **(B)** The correlation of IGF2BP2 and IGF2BP3 expressions with immune cell infiltration in patients with HNSCC. **(C, D)** Protein expression of IGF2BP2 and IGF2BP3 in oral simple epithelial hyperplasia, OED and OSCC increased gradually.

To analyze the expression of 23 methylation regulators in the cancer genome atlas (TCGA) in HNSCC and normal tissues, we searched the GEPIA database and found that only the m6A methylation regulators IGF2BP2 and IGF2BP3 had significant differences in expression in HNSCC than in normal tissues (*P*<0.05) (**Figure 1C**), which was consistent with the results from the Oncomine database. However, there was no significant difference between IGF2BP2 and IGF2BP3 in different stages of HNSCC (*P*>0.05) (**Figure 1D**). Infiltrating immune cells are an important part of the tumor microenvironment and are closely related to tumor progression. Furthermore, we explored the relationship between the expression of m6A methylation regulators and the level of immune infiltration. The results showed that the expression of IGF2BP2 was significantly positively correlated with tumor purity (cor=0.113, *P*=1.19e-02), and was significantly negatively correlated with the infiltration levels of B cells (cor=-0.249, *P*=3.89e-08) and CD8+ T cells (cor=-0.225, *P*=1.91e-08). And the expression of IGF2BP3 was significantly correlated with tumor purity (cor=0.094, *P*=4.03e-02) and CD4+ T cells (cor=0.19, *P*=2.07e-05) (**Figure 2A**).

To obtain the mutation frequency of m6A methylation regulators in HNSCC, we analyzed and integrated the cBioPortal database. The m6A methylation regulators were found to have various degrees of genetic variation in HNSCC (**Figure 2B**), and the most common variation was amplification. Among them, IGF2BP2 had the highest genetic variation frequency (14%), followed by KIAA1429 (5%) and YTHDF3 (4%). The frequencies of other regulators were less than 3%, indicating that changes in the expression levels of these regulators were not caused by genetic alterations.

### Immunohistochemical analysis of m6A methylation regulators IGF2BP2 and IGF2BP3

Combined with the m6A methylation regulator expression profile and prognosis of patients with HNSCC, we selected IGF2BP2 and IGF2BP3 to verify their expression in OSCC and OED. We examined IGF2BP2 and IGF2BP3 expression of 69 OSCC patients (A total of 236 tissue sections) from our department by IHC staining. The results showed that both IGF2BP2 and IGF2BP3 were expressed in the cytoplasm of squamous epithelial cells (**Figures 2C, D**).

**Figure 3:**
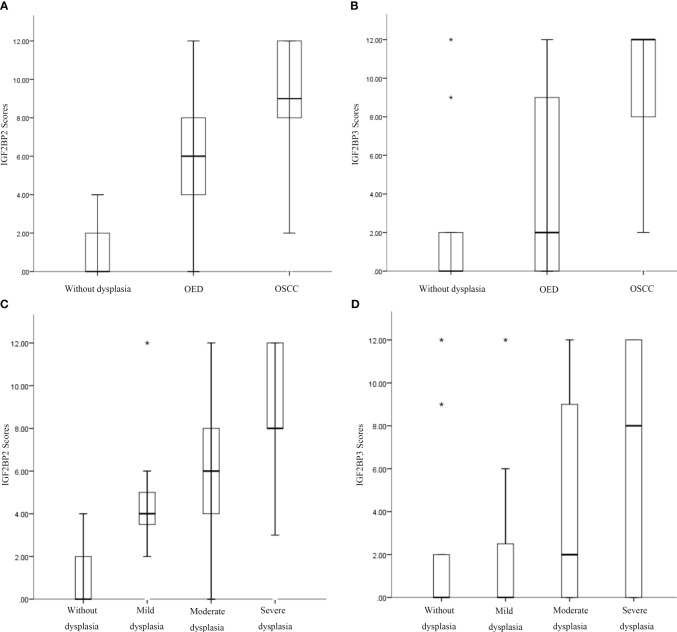
Increased expression of IGF2BP2 and IGF2BP3 was associated with the occurrence of oral squamous cell carcinoma (OSCC), oral epithelial dysplasia (OED), and progression of OED. **(A, B)** Immunohistochemical semiquantitative results of IGF2BP2 and IGF2BP3 expression in oral epithelial hyperplasia, OED, and OSCC. **(C, D)** Immunohistochemical semiquantitative results of IGF2BP2 and IGF2BP3 in epithelial hyperplasia, mild/moderate/severe epithelial dysplasia. *:extreme value.

According to the semi-quantitative results of IHC, we found that the expression of IGF2BP2 increased gradually in epithelial hyperplasia, epithelial dysplasia and squamous cell carcinoma (SCC) (all *P*<0.01) (**Figure 3A**, **Table 3**). And the expression of IGF2BP2 increased in epithelial hyperplasia, mild dysplasia, moderate dysplasia and severe dysplasia (*P*<0.01) (**Figure 3C**). Besides, the expression of IGF2BP3 was increased in epithelial hyperplasia, epithelial dysplasia and SCC (all *P*<0.05) (**Figure 3B**, **Table 3**). However, there was no statistical significance for IGF2BP3 between epithelial hyperplasia and mild dysplasia (*P*>0.05) (**Figure 3D**, **Table 3**).

**Table 3:** Differential expression of IGF2BP2 and IGF2BP3 in epithelial hyperplasia, dysplasia and SCC*.

Clinicopathological diagnosis	*p*	
	IGF2BP2	IGF2BP3
**hyperplasia-dysplasia**	0.000	0.000
**Dysplasia-SCC**	0.000	0.000
**hyperplasia-mild dysplasia**	0.000	0.511
**mild dysplasia-moderate dysplasia**	0.003	0.000
**moderate dysplasia-severe dysplasia**	0.000	0.018

SCC, squamous cell carcinoma.

There was no significant difference in immunohistochemical semiquantitative scores of IGF2BP2 and IGF2BP3 in the epithelial hyperplasia, mild dysplasia, moderate dysplasia and severe dysplasia groups (*P*>0.05). Moreover, the expression of IGF2BP2 and IGF2BP3 were shown moderate correlation (r=0.490, *P*<0.001).

The relationship between the expression of IGF2BP2 and IGF2BP3 in the initial biopsy samples of 69 patients and the clinicopathological characteristics of these patients was analyzed. The results showed that there was no significant difference in the expression of IGF2BP2 and IGF2BP3 among different genders, different ages or smoking/drinking (*P*>0.05). However, they were significantly correlated with pathological diagnosis in the initial biopsy (*P*<0.05) (**Tables 4**, **5**).

**Table 4:** Association between the clinicopathological characteristics and IGF2BP2 expression in 109 cases of OSCC and OED.

Characteristic		IGF2BP2			P*
	n	Negative	Weak Positive	Strong Positive	
Gender					
** Female**	62	22	18	22	0.490
** Male**	47	21	14	12	
Age(years)					
** ≤40**	14	8	4	2	0.380
** 40-60**	56	20	19	17	
** ≥60**	39	15	9	15	
Locations					
** Tongue**	55	18	18	19	0.060
** Buccal**	23	14	4	5	
** Gingiva**	19	9	7	3	
** Other**	12	2	3	7	
Initial diagnosis					
** Epithelial hyperplasia**	28	27	1	0	0.000
** Mild dysplasia**	24	8	14	2	
** Moderate dysplasia**	33	8	11	14	
** Severe dysplasia**	24	0	6	18	
Smoking					
** Yes**	28	13	8	7	0.626
** No**	81	30	24	27	
Drinking					
** Yes**	23	11	5	7	0.577
** No**	86	32	27	27	

* *P* < 0.05 was considered statistically significant.

**Table 5:** Association between the clinicopathological characteristics and IGF2BP3 expression in 109 cases of OSCC and OED.

Characteristic	IGF2BP3				P^*^
	n	Negative	Weak Positive	Strong Positive	
Sex					
** Female**	62	37	10	15	0.497
** Male**	47	31	4	12
Age(years)					
** ≤40**	14	10	2	2	0.895
** 40-60**	56	35	7	14
** ≥60**	39	23	5	11
Locations					
** Tongue**	55	32	10	13	0.119
** Buccal**	23	19	0	4
** Gingiva**	19	12	1	6
** Other**	12	5	3	4
Initial diagnosis					
** Epithelial hyperplasia**	28	25	1	2	0.002
** Mild dysplasia**	24	18	3	3
** Moderate dysplasia**	33	17	5	11
** Severe dysplasia**	24	8	5	11
Smoking					
** Yes**	28	19	0	9	0.054
** No**	81	49	14	18
Drinking					
** Yes**	23	15	0	8	0.084
** No**	86	53	14	19

* P < 0.05 was considered statistically significant.

## Discussion

M6A methylation is one of the significant factors in the occurrence and development of tumors. Reported studies have shown that the m6A write protein METTL3 can recognize the m6A methylation site on the 3’-UTR of B-cell-specific Moloney murine leukemia virus insertion site 1 (BMI1), bind to the m6A read protein IGF2BP1, promote the translation of BMI1 and induce the proliferation and metastasis of OSCC thereby (15). However, at present, there is no comprehensive analysis of the relationship between m6A methylation regulators and tumor progression of HNSCC, especially its role in oral mucosal malignant transformation.

Differential expression analysis showed that there were differences between oral epithelial hyperplasia and epithelial dysplasia (16). The positive rate of P53 was gradually increasing also indicated that the degree of malignancy was increasing (17). Through bioinformatics analysis, we found that high expressions of 7 m6A methylation regulators including IGF2BP2 and IGF2BP3 were associated with poor prognosis. Therefore, IGF2BP2 and IGF2BP3 were selected to be explored in OSCC and OED tissues. It was found that both proteins were highly expressed in OSCC, and their expression increased gradually in oral epithelial hyperplasia, OED, and OSCC.

The insulin-like growth factor 2 mRNA-binding proteins family consists of three restriction enzymes: IGF2BP1, IGF2BP2, and IGF2BP3, which are mainly responsible for regulating the localization, translation, and stability of m6A methylated mRNAs. IGF2BPs exert oncogenic effects by stabilizing m6A-modified mRNAs of oncogenic targets and promoting tumorigenesis (18, 19).

IGF2BP2 is located on chromosome 3q27.2, which binds to the 5’ UTR of insulin-like growth factor 2 mRNA and regulates its translation (20). This study showed that the expression of IGF2BP2 increased in epithelial hyperplasia, OED, and OSCC, and gradually increased with the severity of dysplasia. However, there was no significant difference in expression between epithelial hyperplasia and mild epithelial dysplasia between the progressive and stable groups. Previous studies of IGF2BP2 mainly focused on its role as an RNA-binding protein regulating multiple biological processes. For instance, lncRNA HOTAIR inhibits IGF2BP2 and regulates the growth and invasion of colon cancer (21). In recent years, researchers are more tend to regard IGF2BP2 as a reading protein of m6A to regulate the occurrence and development of tumors. For instance, METTL3 stabilized the expression of SOX2 through an m6A-IGF2BP2-dependent mechanism in colon cancer (22). In a study on esophageal adenocarcinoma, IGF2BP2 was found to be more expressed in metastatic lesions than in primary tumors (23).

IGF2BP3 is located on chromosome 7p15.3 and is a carcinoembryonic protein, which is mainly expressed in the embryonic stage and has a lower expression in adult tissues (24). Most of the studies on IGF2BP3 were analyzed by immunohistochemical methods, and several studies in OSCC confirmed that the expression of IGF2BP3 was up-regulated in OSCC (25, 26). In this study, the expression of IGF2BP3 was positive in epithelial hyperplasia, OED, and OSCC. As an m6A methylation reader protein, IGF2BP3 is also involved in regulating the occurrence and development of tumors. In laryngeal carcinoma, RBM15 and IGF2BP3 are involved in m6A methylation modification of TMBIM6, thereby regulating the expression of TMBIM6 in laryngeal squamous cell carcinoma (LSCC) (27). And some studies have shown that the knockout of IGF2BP3 can inhibit DNA replication and angiogenesis in the S phase of the cell cycle by reading the m6A modification of CCND1 and vascular endothelial growth factor (VEGF) respectively in colon cancer (28).

## Conclusion

We aimed to predict the cancer risk of OED from the perspective of epigenetics. This study suggested that m6A methylation regulators played an important role in head and neck tumor progression and demonstrates that IGF2BP2 and IGF2BP3 were prognostic indicators and potential biomarkers for immunotherapy in OSCC and OED. These findings may provide new strategies for the diagnosis and treatment of OSCC and OED.

## Data availability statement

The raw data supporting the conclusions of this article will be made available by the authors, without undue reservation.

## Ethics statement

The studies involving human participants were reviewed and approved by The Biomedical Ethics Committee of the Department of Peking University (No. PKUSSIRB-201948111).

## Author contributions

All authors listed have made a substantial, direct, and intellectual contribution to the work and approved it for publication.
