# Supplementary material for: Expression and significance of m6A-RNA-methylation in oral cancer and precancerous lesion
Source: Front Oncol. 2023 Jan 30;13:1013054. doi: 10.3389/fonc.2023.1013054 (PMC9923020; doi:10.3389/fonc.2023.1013054)
Supplement: Supplementary file 1 [file DataSheet_1.pdf]

# Oncomine database

## METTL3

| Analysis Type by Cancer | Cancer vs. Normal | | Cancer vs. Cancer | | | | Cancer Subtype Analysis | | | | | | | | | Cancer vs. Baseline (DNA only) | Pathway and Drug | | Outlier | |
|---|---|---|---|---|---|---|---|---|---|---|---|---|---|---|---|---|---|---|---|---|
| | | | Cancer Histology | | Multi-cancer | | Clinical Outcome | Metastasis vs. Primary | Molecular Subtype: Biomarker | Molecular Subtype: Mutation | Pathology Subtype: Grade | Pathology Subtype: Stage | Patient Treatment Response | Recurrence vs. Primary | Other | | Drug Sensitivity | Perturbation | | |
| Bladder Cancer | 1 | | | | | | | | | | | | | | | | | | | 3 |
| Brain and CNS Cancer | 2 | | | | | | | | | | | | | | | | | | | 5 |
| Breast Cancer | | 1 | | | 1 | | | | | | | | | | | | | | 4 | 18 |
| Cervical Cancer | | | | | | | | | | | | | | | | | | | | 2 |
| Colorectal Cancer | 4 | | 2 | 2 | | | | | | | | | | | | | | | 6 | 7 |
| Esophageal Cancer | | | | | | | | | | | | | | | | | | | | 1 |
| Gastric Cancer | | | | | | | | | | | | | | | | | | | 1 | 2 |
| Head and Neck Cancer | 1 | | | | | | | | | | | | | | | | | | 4 | 7 |
| Kidney Cancer | | | 2 | 1 | | | | | | | | | | | | | | | | 5 |
| Leukemia | | 2 | 3 | 3 | 2 | | | | | | | | | | | | | | 2 | 11 |
| Liver Cancer | | | | | | 1 | | | | | | | | | | | | | 3 | 1 |
| Lung Cancer | | | | | | | | | | | | | | | | | | | 4 | 10 |
| Lymphoma | 4 | 2 | | 1 | 1 | | | | | | | | | | | | | | | 9 |
| Melanoma | | | | | | | | | | | | | | | | | | | 2 | 3 |
| Myeloma | | | | | | | | | | | | | | | | | | | 2 | 3 |
| Other Cancer | | | | | | | | | | | | | | | | | | | | 9 |
| Ovarian Cancer | | 1 | | | | | | | | | | | | | | | | | | 5 |
| Pancreatic Cancer | | | | | | 1 | | | | | | | | | | | | | 1 | 2 |
| Prostate Cancer | 1 | | | | 1 | | | | | | | | | | | | | | | 6 |
| Sarcoma | 4 | | 2 | 1 | | | | | | | | | | | | | | | 2 | 8 |
| Significant Unique Analyses | 17 | 5 | 9 | 8 | 4 | 2 | | | | | | | | | | | | | 31 | 109 |
| Total Unique Analyses | 280 | | 480 | | 159 | | | | | | | | | | | | | | 686 | |

## METTL14

| Analysis Type by Cancer | Cancer vs. Normal | | Cancer vs. Cancer | | | | Cancer Subtype Analysis | | | | | | | | | Cancer vs. Baseline (DNA only) | Pathway and Drug | | Outlier | |
|---|---|---|---|---|---|---|---|---|---|---|---|---|---|---|---|---|---|---|---|---|
| | | | Cancer Histology | | Multi-cancer | | Clinical Outcome | Metastasis vs. Primary | Molecular Subtype: Biomarker | Molecular Subtype: Mutation | Pathology Subtype: Grade | Pathology Subtype: Stage | Patient Treatment Response | Recurrence vs. Primary | Other | | Drug Sensitivity | Perturbation | | |
| Bladder Cancer | | | | | | | | | | | | | | | | | | | 1 | 1 |
| Brain and CNS Cancer | | | | | | | | | | | | | | | | | | | | |
| Breast Cancer | | 1 | | | | | | | | | | | | | | | | | 1 | 6 |
| Cervical Cancer | 1 | | | | | | | | | | | | | | | | | | | 1 |
| Colorectal Cancer | | | | | | | | | | | | | | | | | | | 1 | 3 |
| Esophageal Cancer | | | | | | | | | | | | | | | | | | | | |
| Gastric Cancer | | | | | | | | | | | | | | | | | | | 1 | |
| Head and Neck Cancer | | | | | | | | | | | | | | | | | | | 1 | 4 |
| Kidney Cancer | | | | | | | | | | | | | | | | | | | | 2 |
| Leukemia | | | | | | | | | | | | | | | | | | | 1 | 4 |
| Liver Cancer | | | | | | | | | | | | | | | | | | | | 1 |
| Lung Cancer | | | | | | | | | | | | | | | | | | | | 1 |
| Lymphoma | | | | | | | | | | | | | | | | | | | 2 | 6 |
| Melanoma | | | | | | | | | | | | | | | | | | | | |
| Myeloma | | | | | | | | | | | | | | | | | | | | 1 |
| Other Cancer | | | | | | | | | | | | | | | | | | | 1 | 2 |
| Ovarian Cancer | | | | | | | | | | | | | | | | | | | | 2 |
| Pancreatic Cancer | | | | | | | | | | | | | | | | | | | 1 | 1 |
| Prostate Cancer | | | | | | | | | | | | | | | | | | | 2 | 1 |
| Sarcoma | | | | | | | | | | | | | | | | | | | | 1 |
| Significant Unique Analyses | 1 | 1 | | | | | | | | | | | | | | | | | 12 | 33 |
| Total Unique Analyses | 170 | | 281 | | 67 | | | | | | | | | | | | | | 372 | |

# METTL16

| Analysis Type by Cancer | Cancer vs. Normal | | Cancer vs. Cancer | | | | Cancer Subtype Analysis | | | | | | | | | Cancer vs. Baseline (DNA only) | Pathway and Drug | | Outlier | |
|---|---|---|---|---|---|---|---|---|---|---|---|---|---|---|---|---|---|---|---|---|
| | | | Cancer Histology | | Multi-cancer | | Clinical Outcome | Metastasis vs. Primary | Molecular Subtype: Biomarker | Molecular Subtype: Mutation | Pathology Subtype: Grade | Pathology Subtype: Stage | Patient Treatment Response | Recurrence vs. Primary | Other | | Drug Sensitivity | Perturbation | | |
| Bladder Cancer | | | | | | | | | | | | | | | | | | | 1 | |
| Brain and CNS Cancer | | 1 | 1 | 1 | | | | | | | | | | | | | | | 3 | 4 |
| Breast Cancer | | 2 | 1 | 1 | | | | | | | | | | | | | | | 5 | 12 |
| Cervical Cancer | | | | | | | | | | | | | | | | | | | 1 | 1 |
| Colorectal Cancer | 4 | | | | 1 | | | | | | | | | | | | | | 5 | 9 |
| Esophageal Cancer | | | | | | | | | | | | | | | | | | | | 4 |
| Gastric Cancer | | | | | 1 | | | | | | | | | | | | | | 2 | 2 |
| Head and Neck Cancer | | | | | | | | | | | | | | | | | | | 2 | 6 |
| Kidney Cancer | | 1 | 4 | 3 | | | | | | | | | | | | | | | 3 | 5 |
| Leukemia | 1 | 3 | | | 1 | | | | | | | | | | | | | | 3 | 11 |
| Liver Cancer | | | | | | | | | | | | | | | | | | | 2 | 1 |
| Lung Cancer | | | | | | | | | | | | | | | | | | | 4 | 4 |
| Lymphoma | | 1 | | | | | | | | | | | | | | | | | 4 | 7 |
| Melanoma | | | | | | | | | | | | | | | | | | | 3 | 1 |
| Myeloma | | | | | | | | | | | | | | | | | | | 3 | 2 |
| Other Cancer | | | | | | | | | | | | | | | | | | | 5 | 4 |
| Ovarian Cancer | | | | | | | | | | | | | | | | | | | | 3 |
| Pancreatic Cancer | | | | | | | | | | | | | | | | | | | 4 | 4 |
| Prostate Cancer | | | | | | | | | | | | | | | | | | | 1 | 2 |
| Sarcoma | | | 1 | | | | | | | | | | | | | | | | 5 | 4 |
| Significant Unique Analyses | 5 | 8 | 7 | 5 | 3 | | | | | | | | | | | | | | 53 | 80 |
| Total Unique Analyses | 273 | | 465 | | 135 | | | | | | | | | | | | | | 624 | |

# WTAP

| Analysis Type by Cancer | Cancer vs. Normal | | Cancer vs. Cancer | | | | Cancer Subtype Analysis | | | | | | | | | Cancer vs. Baseline (DNA only) | Pathway and Drug | | Outlier | |
|---|---|---|---|---|---|---|---|---|---|---|---|---|---|---|---|---|---|---|---|---|
| | | | Cancer Histology | | Multi-cancer | | Clinical Outcome | Metastasis vs. Primary | Molecular Subtype: Biomarker | Molecular Subtype: Mutation | Pathology Subtype: Grade | Pathology Subtype: Stage | Patient Treatment Response | Recurrence vs. Primary | Other | | Drug Sensitivity | Perturbation | | |
| Bladder Cancer | | | | | | 1 | | | | | | | | | | | | | 3 | 3 |
| Brain and CNS Cancer | 2 | | 1 | 1 | | | | | | | | | | | | | | | 2 | 9 |
| Breast Cancer | 2 | 2 | 2 | 1 | | 1 | | | | | | | | | | | | | 7 | 27 |
| Cervical Cancer | | | | | | | | | | | | | | | | | | | 1 | 2 |
| Colorectal Cancer | | 2 | | | | | | | | | | | | | | | | | 7 | 17 |
| Esophageal Cancer | | 1 | 1 | 1 | | | | | | | | | | | | | | | | 4 |
| Gastric Cancer | | | | | | | | | | | | | | | | | | | 3 | 6 |
| Head and Neck Cancer | 1 | | | | | | | | | | | | | | | | | | 5 | 11 |
| Kidney Cancer | 2 | | 3 | 3 | | | | | | | | | | | | | | | 4 | 3 |
| Leukemia | 1 | 3 | | | 6 | | | | | | | | | | | | | | 7 | 15 |
| Liver Cancer | | | 1 | 1 | | | | | | | | | | | | | | | 1 | 3 |
| Lung Cancer | | | 1 | 1 | | 1 | | | | | | | | | | | | | 2 | 13 |
| Lymphoma | 1 | 3 | | | 1 | | | | | | | | | | | | | | 3 | 15 |
| Melanoma | | | | | | 1 | | | | | | | | | | | | | 2 | 5 |
| Myeloma | | | | | | | | | | | | | | | | | | | 2 | 8 |
| Other Cancer | 3 | | | | | | | | | | | | | | | | | | 3 | 9 |
| Ovarian Cancer | | 2 | | | | 1 | | | | | | | | | | | | | 2 | 6 |
| Pancreatic Cancer | 1 | | | | | 1 | | | | | | | | | | | | | 4 | 4 |
| Prostate Cancer | | 1 | | | 1 | | | | | | | | | | | | | | 4 | 9 |
| Sarcoma | 2 | 1 | 1 | 1 | | | | | | | | | | | | | | | 3 | 9 |
| Significant Unique Analyses | 14 | 15 | 10 | 9 | 7 | 6 | | | | | | | | | | | | | 62 | 165 |
| Total Unique Analyses | 351 | | 573 | | 181 | | | | | | | | | | | | | | 782 | |

## RBM15

| Analysis Type by Cancer | Cancer vs. Normal | | Cancer vs. Cancer | | | | Cancer Subtype Analysis | | | | | | | | | Cancer vs. Baseline (DNA only) | Pathway and Drug | | Outlier | |
|---|---|---|---|---|---|---|---|---|---|---|---|---|---|---|---|---|---|---|---|---|
| | | | Cancer Histology | | Multi-cancer | | Clinical Outcome | Metastasis vs. Primary | Molecular Subtype: Biomarker | Molecular Subtype: Mutation | Pathology Subtype: Grade | Pathology Subtype: Stage | Patient Treatment Response | Recurrence vs. Primary | Other | | Drug Sensitivity | Perturbation | | |
| Bladder Cancer | | | | | | | | | | | | | | | | | | | 1 | 1 |
| Brain and CNS Cancer | 2 | | | | | | | | | | | | | | | | | | 4 | 6 |
| Breast Cancer | | | | | | | | | | | | | | | | | | | 5 | 9 |
| Cervical Cancer | 2 | | | | | | | | | | | | | | | | | | 2 | 2 |
| Colorectal Cancer | | | | | | | | | | | | | | | | | | | 5 | 10 |
| Esophageal Cancer | 1 | | | | | | | | | | | | | | | | | | 1 | 3 |
| Gastric Cancer | 1 | | | | | | | | | | | | | | | | | | 3 | 3 |
| Head and Neck Cancer | | | | | | | | | | | | | | | | | | | | 7 |
| Kidney Cancer | | 1 | | | | | | | | | | | | | | | | | 1 | 1 |
| Leukemia | | 1 | | | | | | | | | | | | | | | | | 4 | 11 |
| Liver Cancer | | | | | | | | | | | | | | | | | | | 1 | 1 |
| Lung Cancer | | | | | | | | | | | | | | | | | | | 2 | 9 |
| Lymphoma | 3 | | | 1 | | | | | | | | | | | | | | | 7 | 9 |
| Melanoma | 1 | | | | | | | | | | | | | | | | | | 2 | 3 |
| Myeloma | | | | | | | | | | | | | | | | | | | 3 | 4 |
| Other Cancer | 2 | | | | | | | | | | | | | | | | | | 3 | 3 |
| Ovarian Cancer | | | | | | | | | | | | | | | | | | | 2 | 2 |
| Pancreatic Cancer | | | | | | | | | | | | | | | | | | | 2 | 4 |
| Prostate Cancer | | | | | | | | | | | | | | | | | | | 7 | 4 |
| Sarcoma | 2 | | 4 | 2 | | | | | | | | | | | | | | | 3 | 5 |
| Significant Unique Analyses | 13 | 2 | 4 | 3 | | | | | | | | | | | | | | | 55 | 94 |
| Total Unique Analyses | 286 | | 491 | | 133 | | | | | | | | | | | | | | 670 | |

## RBM15B

| Analysis Type by Cancer | Cancer vs. Normal | | Cancer vs. Cancer | | | | Cancer Subtype Analysis | | | | | | | | | Cancer vs. Baseline (DNA only) | Pathway and Drug | | Outlier | |
|---|---|---|---|---|---|---|---|---|---|---|---|---|---|---|---|---|---|---|---|---|
| | | | Cancer Histology | | Multi-cancer | | Clinical Outcome | Metastasis vs. Primary | Molecular Subtype: Biomarker | Molecular Subtype: Mutation | Pathology Subtype: Grade | Pathology Subtype: Stage | Patient Treatment Response | Recurrence vs. Primary | Other | | Drug Sensitivity | Perturbation | | |
| Bladder Cancer | | | | | 1 | | | | | | | | | | | | | | | 3 |
| Brain and CNS Cancer | | | | | | | | | | | | | | | | | | | 3 | 1 |
| Breast Cancer | | 1 | | | | | | | | | | | | | | | | | 3 | 10 |
| Cervical Cancer | | | | | | | | | | | | | | | | | | | | 4 |
| Colorectal Cancer | | | | | | 1 | | | | | | | | | | | | | | 5 |
| Esophageal Cancer | | | | | | | | | | | | | | | | | | | | 1 |
| Gastric Cancer | | | | | | | | | | | | | | | | | | | | 1 |
| Head and Neck Cancer | | | | | | | | | | | | | | | | | | | 2 | 4 |
| Kidney Cancer | | | 1 | 2 | | | | | | | | | | | | | | | 3 | 3 |
| Leukemia | 1 | | | | | | | | | | | | | | | | | | 3 | 9 |
| Liver Cancer | | | | | | | | | | | | | | | | | | | | 1 |
| Lung Cancer | | | | | | 2 | | | | | | | | | | | | | 1 | 5 |
| Lymphoma | 1 | | 1 | 1 | | | | | | | | | | | | | | | 3 | 6 |
| Melanoma | | | 1 | | | | | | | | | | | | | | | | 1 | 1 |
| Myeloma | | | | | | | | | | | | | | | | | | | | 2 |
| Other Cancer | 1 | | | | | | | | | | | | | | | | | | 1 | |
| Ovarian Cancer | | | | | 1 | | | | | | | | | | | | | | 4 | 6 |
| Pancreatic Cancer | | | | | | 1 | | | | | | | | | | | | | | 1 |
| Prostate Cancer | | | | | 1 | | | | | | | | | | | | | | 6 | 6 |
| Sarcoma | | | 2 | | | | | | | | | | | | | | | | | 5 |
| Significant Unique Analyses | 3 | 1 | 5 | 3 | 3 | 4 | | | | | | | | | | | | | 30 | 69 |
| Total Unique Analyses | 344 | | 581 | | 185 | | | | | | | | | | | | | | 786 | |

## ZC3H13

| Analysis Type by Cancer | Cancer vs. Normal | | Cancer vs. Cancer | | | | Cancer Subtype Analysis | | | | | | | | | Cancer vs. Baseline (DNA only) | Pathway and Drug | | Outlier | |
|---|---|---|---|---|---|---|---|---|---|---|---|---|---|---|---|---|---|---|---|---|
| | | | Cancer Histology | | Multi-cancer | | Clinical Outcome | Metastasis vs. Primary | Molecular Subtype: Biomarker | Molecular Subtype: Mutation | Pathology Subtype: Grade | Pathology Subtype: Stage | Patient Treatment Response | Recurrence vs. Primary | Other | | Drug Sensitivity | Perturbation | | |
| Bladder Cancer | | | | | | | | | | | | | | | | | | | 3 | 2 |
| Brain and CNS Cancer | | 2 | | | | 1 | | | | | | | | | | | | | 4 | 8 |
| Breast Cancer | | 1 | 1 | 1 | 1 | | | | | | | | | | | | | | 10 | 22 |
| Cervical Cancer | | | | | | | | | | | | | | | | | | | 1 | 1 |
| Colorectal Cancer | 5 | | | | | | | | | | | | | | | | | | 4 | 8 |
| Esophageal Cancer | | 1 | 1 | 1 | | | | | | | | | | | | | | | 4 | 4 |
| Gastric Cancer | | | | | | | | | | | | | | | | | | | 3 | 3 |
| Head and Neck Cancer | 1 | | | | | | | | | | | | | | | | | | 1 | 9 |
| Kidney Cancer | 4 | | 1 | 1 | | | | | | | | | | | | | | | 3 | 8 |
| Leukemia | | | 1 | 1 | 2 | | | | | | | | | | | | | | 6 | 13 |
| Liver Cancer | | 2 | | | | | | | | | | | | | | | | | 1 | 2 |
| Lung Cancer | | 1 | | | | 2 | | | | | | | | | | | | | 2 | 15 |
| Lymphoma | 4 | 4 | | | 1 | 1 | | | | | | | | | | | | | 3 | 11 |
| Melanoma | 1 | | 1 | 1 | 9 | 1 | | | | | | | | | | | | | 4 | 4 |
| Myeloma | | | | | | | | | | | | | | | | | | | | 2 |
| Other Cancer | | | | 1 | | | | | | | | | | | | | | | 1 | 9 |
| Ovarian Cancer | | | | | | 1 | | | | | | | | | | | | | 1 | 6 |
| Pancreatic Cancer | | | | | | | | | | | | | | | | | | | 2 | 4 |
| Prostate Cancer | | 1 | | | 1 | | | | | | | | | | | | | | 5 | 7 |
| Sarcoma | 1 | | 7 | 2 | | | | | | | | | | | | | | | 2 | 9 |
| Significant Unique Analyses | 16 | 12 | 12 | 7 | 14 | 6 | | | | | | | | | | | | | 59 | 139 |
| Total Unique Analyses | 341 | | 572 | | 177 | | | | | | | | | | | | | | 762 | |

## KIAA1429

| Analysis Type by Cancer | Cancer vs. Normal | | Cancer vs. Cancer | | | | Cancer Subtype Analysis | | | | | | | | | Cancer vs. Baseline (DNA only) | Pathway and Drug | | Outlier | |
|---|---|---|---|---|---|---|---|---|---|---|---|---|---|---|---|---|---|---|---|---|
| | | | Cancer Histology | | Multi-cancer | | Clinical Outcome | Metastasis vs. Primary | Molecular Subtype: Biomarker | Molecular Subtype: Mutation | Pathology Subtype: Grade | Pathology Subtype: Stage | Patient Treatment Response | Recurrence vs. Primary | Other | | Drug Sensitivity | Perturbation | | |
| Bladder Cancer | | | | | | 1 | | | | | | | | | | | | | 1 | 1 |
| Brain and CNS Cancer | | 1 | | | | | | | | | | | | | | | | | 1 | 4 |
| Breast Cancer | 2 | | 1 | 1 | 1 | | | | | | | | | | | | | | 10 | 5 |
| Cervical Cancer | 1 | | | | | | | | | | | | | | | | | | 1 | |
| Colorectal Cancer | | | 2 | 2 | | | | | | | | | | | | | | | 3 | 2 |
| Esophageal Cancer | | | | | | | | | | | | | | | | | | | 1 | 1 |
| Gastric Cancer | | | | | | | | | | | | | | | | | | | | 2 |
| Head and Neck Cancer | 3 | | | | | | | | | | | | | | | | | | 2 | 3 |
| Kidney Cancer | | | 1 | | | | | | | | | | | | | | | | | 2 |
| Leukemia | | | | | | | | | | | | | | | | | | | 3 | 3 |
| Liver Cancer | 1 | | 1 | 1 | | | | | | | | | | | | | | | | |
| Lung Cancer | | | | | | | | | | | | | | | | | | | 1 | 3 |
| Lymphoma | | 1 | | 1 | | | | | | | | | | | | | | | 2 | 3 |
| Melanoma | | | | | 1 | | | | | | | | | | | | | | | 2 |
| Myeloma | | | | | | | | | | | | | | | | | | | | 4 |
| Other Cancer | | | | | | | | | | | | | | | | | | | 1 | 3 |
| Ovarian Cancer | | | | | | | | | | | | | | | | | | | 1 | 1 |
| Pancreatic Cancer | | | | | | | | | | | | | | | | | | | 1 | 1 |
| Prostate Cancer | 1 | | | | | 1 | | | | | | | | | | | | | 1 | 2 |
| Sarcoma | | | 1 | | | | | | | | | | | | | | | | 4 | |
| Significant Unique Analyses | 8 | 2 | 5 | 5 | 2 | 2 | | | | | | | | | | | | | 31 | 40 |
| Total Unique Analyses | 204 | | 366 | | 94 | | | | | | | | | | | | | | 442 | |

# FTO

| Analysis Type by Cancer | Cancer vs. Normal | | Cancer vs. Cancer: Cancer Histology | | Cancer vs. Cancer: Multi-cancer | | Cancer Subtype Analysis: Clinical Outcome | Metastasis vs. Primary | Molecular Subtype: Biomarker | Molecular Subtype: Mutation | Pathology Subtype: Grade | Pathology Subtype: Stage | Patient Treatment Response | Recurrence vs. Primary | Other | Cancer vs. Baseline (DNA only) | Pathway and Drug: Drug Sensitivity | Pathway and Drug: Perturbation | Outlier | |
|---|---|---|---|---|---|---|---|---|---|---|---|---|---|---|---|---|---|---|---|---|
| Bladder Cancer | | | | | | | | | | | | | | | | | | | 1 | 2 |
| Brain and CNS Cancer | 1 | | | | | | | | | | | | | | | | | | 1 | 7 |
| Breast Cancer | | 9 | | | 1 | | | | | | | | | | | | | | 14 | 6 |
| Cervical Cancer | | | | | | | | | | | | | | | | | | | 2 | |
| Colorectal Cancer | | | | | | | | | | | | | | | | | | | 3 | 3 |
| Esophageal Cancer | | | | | | | | | | | | | | | | | | | | |
| Gastric Cancer | | | | | | | | | | | | | | | | | | | | 3 |
| Head and Neck Cancer | | | | | | | | | | | | | | | | | | | 3 | 3 |
| Kidney Cancer | 1 | | | 1 | 1 | | | | | | | | | | | | | | 1 | 4 |
| Leukemia | 2 | | 3 | 3 | | | | | | | | | | | | | | | 11 | 13 |
| Liver Cancer | | | | | | | | | | | | | | | | | | | | 7 |
| Lung Cancer | | 1 | | | | | | | | | | | | | | | | | | 5 |
| Lymphoma | 2 | | | | | | | | | | | | | | | | | | 1 | 6 |
| Melanoma | | | | | | | | | | | | | | | | | | | 1 | 2 |
| Myeloma | | | | | | | | | | | | | | | | | | | | 5 |
| Other Cancer | 1 | | 1 | 1 | | | | | | | | | | | | | | | 2 | 11 |
| Ovarian Cancer | | 1 | | | | | | | | | | | | | | | | | 1 | 1 |
| Pancreatic Cancer | | | | | | | | | | | | | | | | | | | | 3 |
| Prostate Cancer | | | | | | | | | | | | | | | | | | | 4 | 6 |
| Sarcoma | 1 | 1 | 1 | 2 | | | | | | | | | | | | | | | | 8 |
| Significant Unique Analyses | 8 | 12 | 5 | 6 | 2 | | | | | | | | | | | | | | 44 | 88 |
| Total Unique Analyses | 418 | | 677 | | 260 | | | | | | | | | | | | | | 900 | |

# ALKBH5

| Analysis Type by Cancer | Cancer vs. Normal | | Cancer vs. Cancer: Cancer Histology | | Cancer vs. Cancer: Multi-cancer | | Cancer Subtype Analysis: Clinical Outcome | Metastasis vs. Primary | Molecular Subtype: Biomarker | Molecular Subtype: Mutation | Pathology Subtype: Grade | Pathology Subtype: Stage | Patient Treatment Response | Recurrence vs. Primary | Other | Cancer vs. Baseline (DNA only) | Pathway and Drug: Drug Sensitivity | Pathway and Drug: Perturbation | Outlier | |
|---|---|---|---|---|---|---|---|---|---|---|---|---|---|---|---|---|---|---|---|---|
| Bladder Cancer | | | | | | | | | | | | | | | | | | | | 3 |
| Brain and CNS Cancer | | | 1 | 1 | | 1 | | | | | | | | | | | | | 5 | 3 |
| Breast Cancer | 1 | 1 | | 1 | | | | | | | | | | | | | | | 7 | 10 |
| Cervical Cancer | | | | | | | | | | | | | | | | | | | 1 | 3 |
| Colorectal Cancer | | | | | | | | | | | | | | | | | | | 3 | 6 |
| Esophageal Cancer | | | | | | | | | | | | | | | | | | | 1 | 2 |
| Gastric Cancer | | | | | | | | | | | | | | | | | | | 1 | 4 |
| Head and Neck Cancer | | | | | | | | | | | | | | | | | | | 1 | 3 |
| Kidney Cancer | | | 1 | 1 | | | | | | | | | | | | | | | 3 | 4 |
| Leukemia | | | | | | | | | | | | | | | | | | | 3 | 7 |
| Liver Cancer | | | | | | | | | | | | | | | | | | | | 5 |
| Lung Cancer | | | | | | | | | | | | | | | | | | | 3 | 6 |
| Lymphoma | | | | | 1 | | | | | | | | | | | | | | 4 | 5 |
| Melanoma | | | | | | 2 | | | | | | | | | | | | | 4 | 2 |
| Myeloma | | | | | | | | | | | | | | | | | | | 4 | 1 |
| Other Cancer | | 8 | 1 | | | | | | | | | | | | | | | | 4 | 7 |
| Ovarian Cancer | | | | | | | | | | | | | | | | | | | | 2 |
| Pancreatic Cancer | | | | | | | | | | | | | | | | | | | 2 | 1 |
| Prostate Cancer | | | | | | | | | | | | | | | | | | | | 1 |
| Sarcoma | | | | | 1 | | | | | | | | | | | | | | 5 | 5 |
| Significant Unique Analyses | 1 | 9 | 3 | 3 | 2 | 3 | | | | | | | | | | | | | 43 | 72 |
| Total Unique Analyses | 312 | | 515 | | 177 | | | | | | | | | | | | | | 598 | |

# YTHDC1

| Analysis Type by Cancer | Cancer vs. Normal | | Cancer vs. Cancer: Cancer Histology | | Cancer vs. Cancer: Multi-cancer | | Cancer Subtype Analysis: Clinical Outcome | Metastasis vs. Primary | Molecular Subtype: Biomarker | Molecular Subtype: Mutation | Pathology Subtype: Grade | Pathology Subtype: Stage | Patient Treatment Response | Recurrence vs. Primary | Other | Cancer vs. Baseline (DNA only) | Pathway and Drug: Drug Sensitivity | Perturbation | Outlier | |
|---|---|---|---|---|---|---|---|---|---|---|---|---|---|---|---|---|---|---|---|---|
| Bladder Cancer | | | | | | | | | | | | | | | | | | | 1 | 2 |
| Brain and CNS Cancer | 2 | 1 | | | | | | | | | | | | | | | | | 5 | 7 |
| Breast Cancer | | 5 | 2 | 2 | | 1 | | | | | | | | | | | | | 14 | 20 |
| Cervical Cancer | | | | | | | | | | | | | | | | | | | 3 | 5 |
| Colorectal Cancer | 5 | | | | | | | | | | | | | | | | | | 11 | 14 |
| Esophageal Cancer | | | | | | | | | | | | | | | | | | | 3 | 4 |
| Gastric Cancer | | | | | 1 | | | | | | | | | | | | | | 3 | 4 |
| Head and Neck Cancer | | | | | | | | | | | | | | | | | | | 4 | 8 |
| Kidney Cancer | 1 | | 3 | 2 | | | | | | | | | | | | | | | 5 | 7 |
| Leukemia | | 2 | 2 | 1 | | | | | | | | | | | | | | | 7 | 18 |
| Liver Cancer | | | 1 | 1 | | | | | | | | | | | | | | | 3 | 4 |
| Lung Cancer | 3 | | 1 | 1 | | | | | | | | | | | | | | | 7 | 14 |
| Lymphoma | 2 | 14 | | | | | | | | | | | | | | | | | 8 | 8 |
| Melanoma | | 1 | | | | | | | | | | | | | | | | | 7 | 8 |
| Myeloma | | | | | | | | | | | | | | | | | | | 3 | 7 |
| Other Cancer | 2 | | 2 | | | | | | | | | | | | | | | | 12 | 11 |
| Ovarian Cancer | | | | | | | | | | | | | | | | | | | 4 | 4 |
| Pancreatic Cancer | | | | | 1 | | | | | | | | | | | | | | 4 | 3 |
| Prostate Cancer | | | | | | | | | | | | | | | | | | | 6 | 9 |
| Sarcoma | | | 4 | 1 | | | | | | | | | | | | | | | 7 | 9 |
| Significant Unique Analyses | 15 | 23 | 14 | 8 | 2 | 1 | | | | | | | | | | | | | 108 | 156 |
| Total Unique Analyses | 337 | | 563 | | 155 | | | | | | | | | | | | | | 758 | |

# YTHDC2

| Analysis Type by Cancer | Cancer vs. Normal | | Cancer vs. Cancer: Cancer Histology | | Cancer vs. Cancer: Multi-cancer | | Cancer Subtype Analysis: Clinical Outcome | Metastasis vs. Primary | Molecular Subtype: Biomarker | Molecular Subtype: Mutation | Pathology Subtype: Grade | Pathology Subtype: Stage | Patient Treatment Response | Recurrence vs. Primary | Other | Cancer vs. Baseline (DNA only) | Pathway and Drug: Drug Sensitivity | Perturbation | Outlier | |
|---|---|---|---|---|---|---|---|---|---|---|---|---|---|---|---|---|---|---|---|---|
| Bladder Cancer | | | | | | | | | | | | | | | | | | | 2 | 2 |
| Brain and CNS Cancer | | | | | | | | | | | | | | | | | | | 3 | 8 |
| Breast Cancer | 1 | | | | | | | | | | | | | | | | | | 4 | 15 |
| Cervical Cancer | | | | | | | | | | | | | | | | | | | 1 | 2 |
| Colorectal Cancer | | | | | | | | | | | | | | | | | | | 3 | 10 |
| Esophageal Cancer | | | | | | | | | | | | | | | | | | | | 4 |
| Gastric Cancer | 1 | | 1 | 2 | | | | | | | | | | | | | | | 1 | 6 |
| Head and Neck Cancer | 1 | | | | | | | | | | | | | | | | | | 6 | 8 |
| Kidney Cancer | | | 1 | 1 | | | | | | | | | | | | | | | 2 | 4 |
| Leukemia | | 1 | 1 | 2 | | | | | | | | | | | | | | | 7 | 15 |
| Liver Cancer | | | | | | | | | | | | | | | | | | | 2 | 4 |
| Lung Cancer | | | | | | | | | | | | | | | | | | | 5 | 13 |
| Lymphoma | | 4 | | | | | | | | | | | | | | | | | 3 | 12 |
| Melanoma | | | | | | | | | | | | | | | | | | | 3 | 6 |
| Myeloma | 1 | | | | | | | | | | | | | | | | | | 1 | 3 |
| Other Cancer | | 3 | 1 | 2 | | | | | | | | | | | | | | | 2 | 8 |
| Ovarian Cancer | | 1 | | | | | | | | | | | | | | | | | | 3 |
| Pancreatic Cancer | | | | | | | | | | | | | | | | | | | 1 | 3 |
| Prostate Cancer | | | | | | | | | | | | | | | | | | | 4 | 10 |
| Sarcoma | 5 | | | | | | | | | | | | | | | | | | 1 | 8 |
| Significant Unique Analyses | 9 | 9 | 4 | 7 | | | | | | | | | | | | | | | 49 | 133 |
| Total Unique Analyses | 331 | | 550 | | 152 | | | | | | | | | | | | | | 748 | |

## YTHDF1

| Analysis Type by Cancer | Cancer vs. Normal | | Cancer vs. Cancer: Cancer Histology | | Cancer vs. Cancer: Multi-cancer | | Cancer Subtype Analysis: Clinical Outcome | Metastasis vs. Primary | Molecular Subtype: Biomarker | Molecular Subtype: Mutation | Pathology Subtype: Grade | Pathology Subtype: Stage | Patient Treatment Response | Recurrence vs. Primary | Other | Cancer vs. Baseline (DNA only) | Pathway and Drug: Drug Sensitivity | Perturbation | Outlier | |
|---|---|---|---|---|---|---|---|---|---|---|---|---|---|---|---|---|---|---|---|---|
| Bladder Cancer | | | | | | 1 | | | | | | | | | | | | | 1 | 2 |
| Brain and CNS Cancer | | | | | 1 | | | | | | | | | | | | | | 3 | 1 |
| Breast Cancer | | | | | 1 | | | | | | | | | | | | | | 10 | 1 |
| Cervical Cancer | | | | | | | | | | | | | | | | | | | 1 | 1 |
| Colorectal Cancer | 2 | | | | | | | | | | | | | | | | | | 3 | |
| Esophageal Cancer | | | | | | | | | | | | | | | | | | | 2 | 2 |
| Gastric Cancer | | | | | | | | | | | | | | | | | | | 3 | |
| Head and Neck Cancer | | | | | | | | | | | | | | | | | | | 1 | |
| Kidney Cancer | | | | | | | | | | | | | | | | | | | 2 | 1 |
| Leukemia | | | | | 1 | | | | | | | | | | | | | | | 2 |
| Liver Cancer | | | | | | | | | | | | | | | | | | | 1 | |
| Lung Cancer | | | | | | | | | | | | | | | | | | | 3 | 2 |
| Lymphoma | | 2 | 1 | 1 | | | | | | | | | | | | | | | 2 | |
| Melanoma | | | | | | | | | | | | | | | | | | | | 1 |
| Myeloma | | | | | | | | | | | | | | | | | | | 1 | |
| Other Cancer | | | | | | | | | | | | | | | | | | | 9 | 1 |
| Ovarian Cancer | | | | | | | | | | | | | | | | | | | 2 | 1 |
| Pancreatic Cancer | | | 1 | 1 | | | | | | | | | | | | | | | 2 | 1 |
| Prostate Cancer | | | | | | | | | | | | | | | | | | | 4 | 1 |
| Sarcoma | | | | | | | | | | | | | | | | | | | 3 | |
| Significant Unique Analyses | 2 | 2 | 2 | 2 | 3 | 1 | | | | | | | | | | | | | 46 | 16 |
| Total Unique Analyses | 180 | | 267 | | 110 | | | | | | | | | | | | | | 300 | |

## YTHDF2

| Analysis Type by Cancer | Cancer vs. Normal | | Cancer vs. Cancer: Cancer Histology | | Cancer vs. Cancer: Multi-cancer | | Cancer Subtype Analysis: Clinical Outcome | Metastasis vs. Primary | Molecular Subtype: Biomarker | Molecular Subtype: Mutation | Pathology Subtype: Grade | Pathology Subtype: Stage | Patient Treatment Response | Recurrence vs. Primary | Other | Cancer vs. Baseline (DNA only) | Pathway and Drug: Drug Sensitivity | Perturbation | Outlier | |
|---|---|---|---|---|---|---|---|---|---|---|---|---|---|---|---|---|---|---|---|---|
| Bladder Cancer | | | | | | | | | | | | | | | | | | | 1 | 3 |
| Brain and CNS Cancer | 1 | 1 | | | | | | | | | | | | | | | | | 1 | 5 |
| Breast Cancer | 2 | | | | | 2 | | | | | | | | | | | | | | 10 |
| Cervical Cancer | 1 | | | | | | | | | | | | | | | | | | 1 | 2 |
| Colorectal Cancer | | | | | | | | | | | | | | | | | | | 1 | 7 |
| Esophageal Cancer | | | | | | | | | | | | | | | | | | | | 3 |
| Gastric Cancer | | | | | | | | | | | | | | | | | | | | |
| Head and Neck Cancer | 1 | | | | | | | | | | | | | | | | | | 2 | 4 |
| Kidney Cancer | 1 | | | 1 | | | | | | | | | | | | | | | 1 | 5 |
| Leukemia | | | | | 1 | | | | | | | | | | | | | | | 9 |
| Liver Cancer | | | | | | | | | | | | | | | | | | | | 3 |
| Lung Cancer | | | | | | 1 | | | | | | | | | | | | | 3 | 3 |
| Lymphoma | | | | | | | | | | | | | | | | | | | 2 | 6 |
| Melanoma | | | | | 1 | | | | | | | | | | | | | | | 2 |
| Myeloma | | | | | | | | | | | | | | | | | | | 1 | 3 |
| Other Cancer | 2 | | | | | | | | | | | | | | | | | | 1 | 6 |
| Ovarian Cancer | | | | | | | | | | | | | | | | | | | 1 | 4 |
| Pancreatic Cancer | | | | | | | | | | | | | | | | | | | 1 | 2 |
| Prostate Cancer | | | | | 1 | | | | | | | | | | | | | | 3 | 5 |
| Sarcoma | | | | | | | | | | | | | | | | | | | 1 | 6 |
| Significant Unique Analyses | 8 | 1 | | 1 | 3 | 3 | | | | | | | | | | | | | 19 | 82 |
| Total Unique Analyses | 295 | | 517 | | 142 | | | | | | | | | | | | | | 666 | |

## YTHDF3

| Analysis Type by Cancer | Cancer vs. Normal | | Cancer vs. Cancer | | | | Cancer Subtype Analysis | | | | | | | | | Cancer vs. Baseline (DNA only) | Pathway and Drug | | Outlier | |
|---|---|---|---|---|---|---|---|---|---|---|---|---|---|---|---|---|---|---|---|---|
| | | | Cancer Histology | | Multi-cancer | | Clinical Outcome | Metastasis vs. Primary | Molecular Subtype: Biomarker | Molecular Subtype: Mutation | Pathology Subtype: Grade | Pathology Subtype: Stage | Patient Treatment Response | Recurrence vs. Primary | Other | | Drug Sensitivity | Perturbation | | |
| Bladder Cancer | | | | | | | | | | | | | | | | | | | 1 | 1 |
| Brain and CNS Cancer | 2 | | | | | | | | | | | | | | | | | | 2 | 7 |
| Breast Cancer | | | | | | 1 | | | | | | | | | | | | | 3 | 13 |
| Cervical Cancer | 1 | | 1 | | | | | | | | | | | | | | | | | 2 |
| Colorectal Cancer | | | | | | | | | | | | | | | | | | | 3 | 6 |
| Esophageal Cancer | | | | | | | | | | | | | | | | | | | | 2 |
| Gastric Cancer | | | | | | | | | | | | | | | | | | | 1 | 1 |
| Head and Neck Cancer | 1 | | | | | | | | | | | | | | | | | | 1 | 4 |
| Kidney Cancer | 1 | | | | | 1 | | | | | | | | | | | | | 2 | 1 |
| Leukemia | | | 1 | | | | | | | | | | | | | | | | 2 | 9 |
| Liver Cancer | | | | | | 1 | | | | | | | | | | | | | | 2 |
| Lung Cancer | | | | | | | | | | | | | | | | | | | 2 | 6 |
| Lymphoma | 2 | 1 | | | | | | | | | | | | | | | | | | 8 |
| Melanoma | | | | | 1 | | | | | | | | | | | | | | 1 | 3 |
| Myeloma | | | | | | | | | | | | | | | | | | | | 1 |
| Other Cancer | | | | | | | | | | | | | | | | | | | 1 | 2 |
| Ovarian Cancer | | 1 | 2 | 2 | | | | | | | | | | | | | | | | |
| Pancreatic Cancer | | | | | 1 | | | | | | | | | | | | | | | 6 |
| Prostate Cancer | | | | | 1 | 1 | | | | | | | | | | | | | 3 | 4 |
| Sarcoma | | | 1 | | | | | | | | | | | | | | | | 1 | 7 |
| Significant Unique Analyses | 7 | 2 | 5 | 2 | 3 | 4 | | | | | | | | | | | | | 22 | 77 |
| Total Unique Analyses | 298 | | 518 | | 177 | | | | | | | | | | | | | | 724 | |

## HNRNPA2B1

| Analysis Type by Cancer | Cancer vs. Normal | | Cancer vs. Cancer | | | | Cancer Subtype Analysis | | | | | | | | | Cancer vs. Baseline (DNA only) | Pathway and Drug | | Outlier | |
|---|---|---|---|---|---|---|---|---|---|---|---|---|---|---|---|---|---|---|---|---|
| | | | Cancer Histology | | Multi-cancer | | Clinical Outcome | Metastasis vs. Primary | Molecular Subtype: Biomarker | Molecular Subtype: Mutation | Pathology Subtype: Grade | Pathology Subtype: Stage | Patient Treatment Response | Recurrence vs. Primary | Other | | Drug Sensitivity | Perturbation | | |
| Bladder Cancer | 1 | | | | | | | | | | | | | | | | | | 2 | 2 |
| Brain and CNS Cancer | 5 | | | | | | | | | | | | | | | | | | 6 | 8 |
| Breast Cancer | 1 | 1 | | | | 2 | | | | | | | | | | | | | 4 | 16 |
| Cervical Cancer | 1 | | | | | | | | | | | | | | | | | | 2 | 1 |
| Colorectal Cancer | 2 | | 2 | 2 | 1 | | | | | | | | | | | | | | 3 | 9 |
| Esophageal Cancer | | | | | | | | | | | | | | | | | | | 1 | 3 |
| Gastric Cancer | | | | | | | | | | | | | | | | | | | 5 | 1 |
| Head and Neck Cancer | 5 | | | 1 | | | | | | | | | | | | | | | 4 | 8 |
| Kidney Cancer | | | 1 | 1 | | | | | | | | | | | | | | | 1 | 4 |
| Leukemia | 1 | 1 | 1 | 1 | 2 | | | | | | | | | | | | | | 4 | 15 |
| Liver Cancer | | | | | | | | | | | | | | | | | | | 2 | 4 |
| Lung Cancer | | | 1 | 1 | | | | | | | | | | | | | | | 5 | 11 |
| Lymphoma | 2 | 1 | 1 | 2 | | | | | | | | | | | | | | | 1 | 8 |
| Melanoma | | | | | 1 | | | | | | | | | | | | | | 1 | 1 |
| Myeloma | | | | | | | | | | | | | | | | | | | 1 | 3 |
| Other Cancer | 7 | | 2 | 1 | | | | | | | | | | | | | | | 3 | 7 |
| Ovarian Cancer | | | | | | | | | | | | | | | | | | | | 6 |
| Pancreatic Cancer | 1 | | | | | | | | | | | | | | | | | | 2 | 4 |
| Prostate Cancer | | | | | | | | | | | | | | | | | | | 3 | 8 |
| Sarcoma | 3 | | 1 | | | | | | | | | | | | | | | | | 3 |
| Significant Unique Analyses | 28 | 3 | 9 | 9 | 4 | 2 | | | | | | | | | | | | | 48 | 118 |
| Total Unique Analyses | 349 | | 591 | | 184 | | | | | | | | | | | | | | 800 | |

## HNRNPC

| Analysis Type by Cancer | Cancer vs. Normal | | Cancer vs. Cancer: Cancer Histology | | Cancer vs. Cancer: Multi-cancer | | Cancer Subtype Analysis: Clinical Outcome | Metastasis vs. Primary | Molecular Subtype: Biomarker | Molecular Subtype: Mutation | Pathology Subtype: Grade | Pathology Subtype: Stage | Patient Treatment Response | Recurrence vs. Primary | Other | Cancer vs. Baseline (DNA only) | Pathway and Drug: Drug Sensitivity | Perturbation | Outlier | |
|---|---|---|---|---|---|---|---|---|---|---|---|---|---|---|---|---|---|---|---|---|
| Bladder Cancer | | | | | | 1 | | | | | | | | | | | | | | 5 |
| Brain and CNS Cancer | 4 | | 1 | 1 | | | | | | | | | | | | | | | 3 | 9 |
| Breast Cancer | | 1 | | | | | | | | | | | | | | | | | 7 | 24 |
| Cervical Cancer | | | | | | | | | | | | | | | | | | | 2 | 2 |
| Colorectal Cancer | 2 | | | | | 2 | | | | | | | | | | | | | 6 | 11 |
| Esophageal Cancer | | | | | | | | | | | | | | | | | | | 3 | 3 |
| Gastric Cancer | | | | | 1 | | | | | | | | | | | | | | 1 | 2 |
| Head and Neck Cancer | 2 | | | | | | | | | | | | | | | | | | 4 | 5 |
| Kidney Cancer | 2 | | 1 | 1 | | 1 | | | | | | | | | | | | | 5 | 10 |
| Leukemia | 2 | | 2 | 2 | 1 | | | | | | | | | | | | | | 3 | 22 |
| Liver Cancer | | | | | | | | | | | | | | | | | | | 2 | 3 |
| Lung Cancer | | | | | | 2 | | | | | | | | | | | | | 4 | 13 |
| Lymphoma | 1 | 2 | | | 1 | | | | | | | | | | | | | | 4 | 10 |
| Melanoma | 2 | | | | | | | | | | | | | | | | | | 3 | 6 |
| Myeloma | 2 | | 1 | 1 | | | | | | | | | | | | | | | | 2 |
| Other Cancer | 1 | | | | | | | | | | | | | | | | | | 1 | 12 |
| Ovarian Cancer | | | | | | 1 | | | | | | | | | | | | | 1 | 6 |
| Pancreatic Cancer | | 1 | | | | 1 | | | | | | | | | | | | | 1 | 8 |
| Prostate Cancer | 2 | | | | | | | | | | | | | | | | | | 7 | 8 |
| Sarcoma | 3 | | 2 | 2 | | | | | | | | | | | | | | | 3 | 9 |
| Significant Unique Analyses | 23 | 4 | 7 | 7 | 3 | 8 | | | | | | | | | | | | | 58 | 159 |
| Total Unique Analyses | 347 | | 598 | | 185 | | | | | | | | | | | | | | 812 | |

## HNRNPG

| Analysis Type by Cancer | Cancer vs. Normal | | Cancer vs. Cancer: Cancer Histology | | Cancer vs. Cancer: Multi-cancer | | Cancer Subtype Analysis: Clinical Outcome | Metastasis vs. Primary | Molecular Subtype: Biomarker | Molecular Subtype: Mutation | Pathology Subtype: Grade | Pathology Subtype: Stage | Patient Treatment Response | Recurrence vs. Primary | Other | Cancer vs. Baseline (DNA only) | Pathway and Drug: Drug Sensitivity | Perturbation | Outlier | |
|---|---|---|---|---|---|---|---|---|---|---|---|---|---|---|---|---|---|---|---|---|
| Bladder Cancer | | | | | | 1 | | | | | | | | | | | | | 2 | 4 |
| Brain and CNS Cancer | 5 | | 1 | 1 | | | | | | | | | | | | | | | 2 | 5 |
| Breast Cancer | | 1 | | 1 | | | | | | | | | | | | | | | 10 | 11 |
| Cervical Cancer | | | | | | | | | | | | | | | | | | | 2 | |
| Colorectal Cancer | 5 | | | | | 1 | | | | | | | | | | | | | 5 | 5 |
| Esophageal Cancer | | | | | | | | | | | | | | | | | | | 2 | 3 |
| Gastric Cancer | | | | | | | | | | | | | | | | | | | 2 | 1 |
| Head and Neck Cancer | | | | | | | | | | | | | | | | | | | | 4 |
| Kidney Cancer | 1 | | 1 | | | | | | | | | | | | | | | | 2 | 3 |
| Leukemia | | | | | 1 | | | | | | | | | | | | | | 4 | 12 |
| Liver Cancer | | | | | | | | | | | | | | | | | | | 2 | 2 |
| Lung Cancer | | | 1 | 1 | | 1 | | | | | | | | | | | | | 4 | 5 |
| Lymphoma | 1 | 2 | 1 | 1 | | | | | | | | | | | | | | | 3 | 6 |
| Melanoma | | | | | | 1 | | | | | | | | | | | | | 1 | 1 |
| Myeloma | 1 | | | | | | | | | | | | | | | | | | | 1 |
| Other Cancer | 10 | | | 2 | | | | | | | | | | | | | | | 2 | 3 |
| Ovarian Cancer | | 2 | | | | 1 | | | | | | | | | | | | | 3 | 2 |
| Pancreatic Cancer | | | | | | 1 | | | | | | | | | | | | | 1 | 1 |
| Prostate Cancer | 3 | | | | | | | | | | | | | | | | | | 3 | 9 |
| Sarcoma | 3 | 1 | 1 | 1 | | | | | | | | | | | | | | | 1 | 5 |
| Significant Unique Analyses | 29 | 6 | 5 | 6 | 1 | 6 | | | | | | | | | | | | | 49 | 79 |
| Total Unique Analyses | 349 | | 605 | | 188 | | | | | | | | | | | | | | 812 | |

# FMR1

| Analysis Type by Cancer | Cancer vs. Normal | | Cancer vs. Cancer | | | | Cancer Subtype Analysis | | | | | | | | | Cancer vs. Baseline (DNA only) | Pathway and Drug | | Outlier | |
|---|---|---|---|---|---|---|---|---|---|---|---|---|---|---|---|---|---|---|---|---|
| | | | Cancer Histology | | Multi-cancer | | Clinical Outcome | Metastasis vs. Primary | Molecular Subtype: Biomarker | Molecular Subtype: Mutation | Pathology Subtype: Grade | Pathology Subtype: Stage | Patient Treatment Response | Recurrence vs. Primary | Other | | Drug Sensitivity | Perturbation | | |
| Bladder Cancer | | | 1 | 1 | | | | | | | | | | | | | | | 1 | 1 |
| Brain and CNS Cancer | | 2 | | | 1 | | | | | | | | | | | | | | 3 | 5 |
| Breast Cancer | | | 1 | 1 | | 2 | | | | | | | | | | | | | 3 | 16 |
| Cervical Cancer | 2 | | | | | | | | | | | | | | | | | | | 2 |
| Colorectal Cancer | | | | | | 1 | | | | | | | | | | | | | 7 | 7 |
| Esophageal Cancer | | | 1 | 1 | | | | | | | | | | | | | | | 2 | |
| Gastric Cancer | 1 | | | | | | | | | | | | | | | | | | | 2 |
| Head and Neck Cancer | | | | | 1 | | | | | | | | | | | | | | | 4 |
| Kidney Cancer | | | 1 | 1 | | | | | | | | | | | | | | | 1 | 3 |
| Leukemia | | | 2 | 2 | | | | | | | | | | | | | | | 1 | 11 |
| Liver Cancer | | | | | | | | | | | | | | | | | | | | 3 |
| Lung Cancer | 1 | | | | | | | | | | | | | | | | | | 4 | 5 |
| Lymphoma | | 1 | | | | | | | | | | | | | | | | | 1 | 9 |
| Melanoma | | | | | | | | | | | | | | | | | | | | 2 |
| Myeloma | | | | | | | | | | | | | | | | | | | 1 | 2 |
| Other Cancer | 4 | 1 | | | | | | | | | | | | | | | | | 4 | 8 |
| Ovarian Cancer | 1 | | | 1 | | 1 | | | | | | | | | | | | | 3 | 1 |
| Pancreatic Cancer | | | | | | | | | | | | | | | | | | | | 2 |
| Prostate Cancer | | | | | | | | | | | | | | | | | | | 4 | 8 |
| Sarcoma | 5 | | 2 | | | | | | | | | | | | | | | | 3 | 5 |
| Significant Unique Analyses | 13 | 4 | 8 | 7 | 2 | 4 | | | | | | | | | | | | | 35 | 87 |
| Total Unique Analyses | 352 | | 598 | | 188 | | | | | | | | | | | | | | 800 | |

# LRPPRC

| Analysis Type by Cancer | Cancer vs. Normal | | Cancer vs. Cancer | | | | Cancer Subtype Analysis | | | | | | | | | Cancer vs. Baseline (DNA only) | Pathway and Drug | | Outlier | |
|---|---|---|---|---|---|---|---|---|---|---|---|---|---|---|---|---|---|---|---|---|
| | | | Cancer Histology | | Multi-cancer | | Clinical Outcome | Metastasis vs. Primary | Molecular Subtype: Biomarker | Molecular Subtype: Mutation | Pathology Subtype: Grade | Pathology Subtype: Stage | Patient Treatment Response | Recurrence vs. Primary | Other | | Drug Sensitivity | Perturbation | | |
| Bladder Cancer | | | | | | 1 | | | | | | | | | | | | | 1 | 3 |
| Brain and CNS Cancer | | 2 | | | | 1 | | | | | | | | | | | | | 4 | 1 |
| Breast Cancer | 1 | 1 | 2 | 2 | | | | | | | | | | | | | | | 5 | 14 |
| Cervical Cancer | | | | | | | | | | | | | | | | | | | 2 | 1 |
| Colorectal Cancer | | | | | | | | | | | | | | | | | | | 5 | 8 |
| Esophageal Cancer | | | | | | | | | | | | | | | | | | | 4 | |
| Gastric Cancer | 1 | | | | | | | | | | | | | | | | | | 1 | |
| Head and Neck Cancer | 1 | | | | | | | | | | | | | | | | | | 4 | 2 |
| Kidney Cancer | | | 1 | 2 | | | | | | | | | | | | | | | 3 | 4 |
| Leukemia | 2 | | | | | | | | | | | | | | | | | | 4 | 8 |
| Liver Cancer | | | | | | | | | | | | | | | | | | | 1 | 3 |
| Lung Cancer | | | | | | | | | | | | | | | | | | | 5 | 4 |
| Lymphoma | 1 | | 2 | 4 | | | | | | | | | | | | | | | 5 | 7 |
| Melanoma | | | | | | | | | | | | | | | | | | | 1 | 4 |
| Myeloma | 2 | | | | | | | | | | | | | | | | | | 1 | 5 |
| Other Cancer | 3 | 1 | | 2 | | | | | | | | | | | | | | | 6 | 6 |
| Ovarian Cancer | | | | | 1 | | | | | | | | | | | | | | 3 | 5 |
| Pancreatic Cancer | | | | | | | | | | | | | | | | | | | 1 | 2 |
| Prostate Cancer | 1 | | | | | | | | | | | | | | | | | | 6 | 3 |
| Sarcoma | 2 | | 6 | 3 | | | | | | | | | | | | | | | 4 | 3 |
| Significant Unique Analyses | 14 | 4 | 11 | 11 | 1 | 2 | | | | | | | | | | | | | 63 | 74 |
| Total Unique Analyses | 343 | | 578 | | 185 | | | | | | | | | | | | | | 798 | |

# IGF2BP1

| Analysis Type by Cancer | Cancer vs. Normal | | Cancer vs. Cancer: Cancer Histology | | Cancer vs. Cancer: Multi-cancer | | Cancer Subtype Analysis: Clinical Outcome | Metastasis vs. Primary | Molecular Subtype: Biomarker | Molecular Subtype: Mutation | Pathology Subtype: Grade | Pathology Subtype: Stage | Patient Treatment Response | Recurrence vs. Primary | Other | Cancer vs. Baseline (DNA only) | Pathway and Drug: Drug Sensitivity | Perturbation | Outlier | |
|---|---|---|---|---|---|---|---|---|---|---|---|---|---|---|---|---|---|---|---|---|
| Bladder Cancer | | | | | | | | | | | | | | | | | | | | |
| Brain and CNS Cancer | 3 | | 2 | 2 | | 1 | | | | | | | | | | | | | 6 | 4 |
| Breast Cancer | | 2 | 1 | 1 | | 1 | | | | | | | | | | | | | 13 | 10 |
| Cervical Cancer | | | 1 | | | | | | | | | | | | | | | | 3 | 1 |
| Colorectal Cancer | | 1 | 1 | 1 | | 1 | | | | | | | | | | | | | 16 | 3 |
| Esophageal Cancer | | 1 | 1 | 1 | | | | | | | | | | | | | | | 2 | 1 |
| Gastric Cancer | | | | | 1 | | | | | | | | | | | | | | 7 | 2 |
| Head and Neck Cancer | | | | | | | | | | | | | | | | | | | 7 | |
| Kidney Cancer | | 2 | | 1 | | | | | | | | | | | | | | | 3 | 1 |
| Leukemia | 1 | | | | 2 | | | | | | | | | | | | | | 11 | 2 |
| Liver Cancer | | | 1 | 1 | 2 | | | | | | | | | | | | | | 3 | 1 |
| Lung Cancer | 1 | | 2 | 2 | | | | | | | | | | | | | | | 8 | 1 |
| Lymphoma | | | | | | | | | | | | | | | | | | | 11 | 2 |
| Melanoma | | | | | | | | | | | | | | | | | | | 6 | |
| Myeloma | | | | | | | | | | | | | | | | | | | 1 | 1 |
| Other Cancer | 4 | | 2 | 2 | 1 | | | | | | | | | | | | | | 9 | |
| Ovarian Cancer | | | | | | | | | | | | | | | | | | | 3 | 1 |
| Pancreatic Cancer | | 2 | 1 | 1 | | 1 | | | | | | | | | | | | | 6 | 3 |
| Prostate Cancer | | | | | | 1 | | | | | | | | | | | | | 3 | 1 |
| Sarcoma | | | 2 | 2 | | | | | | | | | | | | | | | 11 | 3 |
| Significant Unique Analyses | 9 | 8 | 14 | 13 | 5 | 5 | | | | | | | | | | | | | 116 | 36 |
| Total Unique Analyses | 211 | | 358 | | 85 | | | | | | | | | | | | | | 436 | |

# IGF2BP2

| Analysis Type by Cancer | Cancer vs. Normal | | Cancer vs. Cancer: Cancer Histology | | Cancer vs. Cancer: Multi-cancer | | Cancer Subtype Analysis: Clinical Outcome | Metastasis vs. Primary | Molecular Subtype: Biomarker | Molecular Subtype: Mutation | Pathology Subtype: Grade | Pathology Subtype: Stage | Patient Treatment Response | Recurrence vs. Primary | Other | Cancer vs. Baseline (DNA only) | Pathway and Drug: Drug Sensitivity | Perturbation | Outlier | |
|---|---|---|---|---|---|---|---|---|---|---|---|---|---|---|---|---|---|---|---|---|
| Bladder Cancer | | | | | 1 | | | | | | | | | | | | | | | |
| Brain and CNS Cancer | 4 | | 2 | 2 | | | | | | | | | | | | | | | 3 | 4 |
| Breast Cancer | | 3 | 2 | 2 | | 3 | | | | | | | | | | | | | 23 | 11 |
| Cervical Cancer | 1 | | 2 | 1 | | | | | | | | | | | | | | | 1 | 2 |
| Colorectal Cancer | 1 | | | | 4 | | | | | | | | | | | | | | 1 | 13 |
| Esophageal Cancer | 6 | | | | 4 | | | | | | | | | | | | | | 2 | 2 |
| Gastric Cancer | | | 1 | 1 | | | | | | | | | | | | | | | 3 | 6 |
| Head and Neck Cancer | 7 | | | | 3 | | | | | | | | | | | | | | 2 | 7 |
| Kidney Cancer | | 3 | 4 | 3 | 4 | | | | | | | | | | | | | | 4 | 5 |
| Leukemia | 2 | 3 | 7 | 7 | | | | | | | | | | | | | | | 6 | 10 |
| Liver Cancer | 4 | | 1 | 1 | 4 | | | | | | | | | | | | | | 3 | 1 |
| Lung Cancer | 2 | | 7 | 6 | 1 | | | | | | | | | | | | | | 3 | 9 |
| Lymphoma | 3 | | | | | 5 | | | | | | | | | | | | | 8 | 3 |
| Melanoma | | | | | 8 | | | | | | | | | | | | | | 2 | 3 |
| Myeloma | | 2 | | | | | | | | | | | | | | | | | 5 | |
| Other Cancer | 4 | 2 | 4 | 4 | 3 | | | | | | | | | | | | | | 7 | 2 |
| Ovarian Cancer | 1 | | 2 | 3 | 5 | | | | | | | | | | | | | | 1 | 5 |
| Pancreatic Cancer | 3 | | | | 4 | | | | | | | | | | | | | | | 2 |
| Prostate Cancer | | | | | | 2 | | | | | | | | | | | | | 4 | 4 |
| Sarcoma | 1 | 3 | 10 | 9 | 2 | 1 | | | | | | | | | | | | | 2 | 6 |
| Significant Unique Analyses | 39 | 16 | 42 | 39 | 40 | 10 | | | | | | | | | | | | | 73 | 90 |
| Total Unique Analyses | 282 | | 491 | | 150 | | | | | | | | | | | | | | 658 | |

# IGF2BP3

| Analysis Type by Cancer | Cancer vs. Normal | | Cancer vs. Cancer | | | | Cancer Subtype Analysis | | | | | | | | | Cancer vs. Baseline (DNA only) | Pathway and Drug | | Outlier | |
|---|---|---|---|---|---|---|---|---|---|---|---|---|---|---|---|---|---|---|---|---|
| | | | Cancer Histology | | Multi-cancer | | Clinical Outcome | Metastasis vs. Primary | Molecular Subtype: Biomarker | Molecular Subtype: Mutation | Pathology Subtype: Grade | Pathology Subtype: Stage | Patient Treatment Response | Recurrence vs. Primary | Other | | Drug Sensitivity | Perturbation | | |
| Bladder Cancer | 1 | | 4 | 4 | | | | | | | | | | | | | | | 3 | 1 |
| Brain and CNS Cancer | 6 | 1 | 9 | 12 | | | | | | | | | | | | | | | 9 | 7 |
| Breast Cancer | | | | 1 | 1 | 7 | | | | | | | | | | | | | 38 | 16 |
| Cervical Cancer | 1 | | 3 | 3 | | | | | | | | | | | | | | | 4 | 2 |
| Colorectal Cancer | | | | 1 | | 1 | | | | | | | | | | | | | 19 | 4 |
| Esophageal Cancer | 1 | | 2 | 2 | 2 | | | | | | | | | | | | | | 2 | 3 |
| Gastric Cancer | 6 | | 3 | 3 | | | | | | | | | | | | | | | 5 | 6 |
| Head and Neck Cancer | 4 | | | | | | | | | | | | | | | | | | 9 | 4 |
| Kidney Cancer | | 1 | 1 | 2 | 1 | | | | | | | | | | | | | | 10 | 4 |
| Leukemia | 4 | 2 | 8 | 7 | | | | | | | | | | | | | | | 16 | 22 |
| Liver Cancer | 3 | | 1 | 1 | 2 | | | | | | | | | | | | | | 6 | |
| Lung Cancer | 13 | | 7 | 8 | 5 | | | | | | | | | | | | | | 9 | 9 |
| Lymphoma | | | 2 | 3 | 1 | | | | | | | | | | | | | | 10 | 10 |
| Melanoma | 1 | | | | 1 | | | | | | | | | | | | | | 4 | 6 |
| Myeloma | | 2 | | | | | | | | | | | | | | | | | 6 | 2 |
| Other Cancer | 4 | | 2 | 2 | 1 | | | | | | | | | | | | | | 12 | 8 |
| Ovarian Cancer | 3 | | 1 | 1 | 1 | | | | | | | | | | | | | | 10 | 1 |
| Pancreatic Cancer | 4 | | | | 4 | | | | | | | | | | | | | | 6 | 3 |
| Prostate Cancer | | | | | | 2 | | | | | | | | | | | | | 10 | 5 |
| Sarcoma | 3 | | 7 | 7 | 1 | | | | | | | | | | | | | | 9 | 6 |
| Significant Unique Analyses | 53 | 6 | 50 | 57 | 19 | 10 | | | | | | | | | | | | | 184 | 104 |
| Total Unique Analyses | 329 | | 559 | | 171 | | | | | | | | | | | | | | 760 | |
