# Supplementary material for: Expression and significance of m6A-RNA-methylation in oral cancer and precancerous lesion
Source: Front Oncol. 2023 Jan 30;13:1013054. doi: 10.3389/fonc.2023.1013054 (PMC9923020; doi:10.3389/fonc.2023.1013054)
Supplement: Supplementary file 2 [file DataSheet_2.pdf]

Kaplan-Meier Plotter database

METTL3

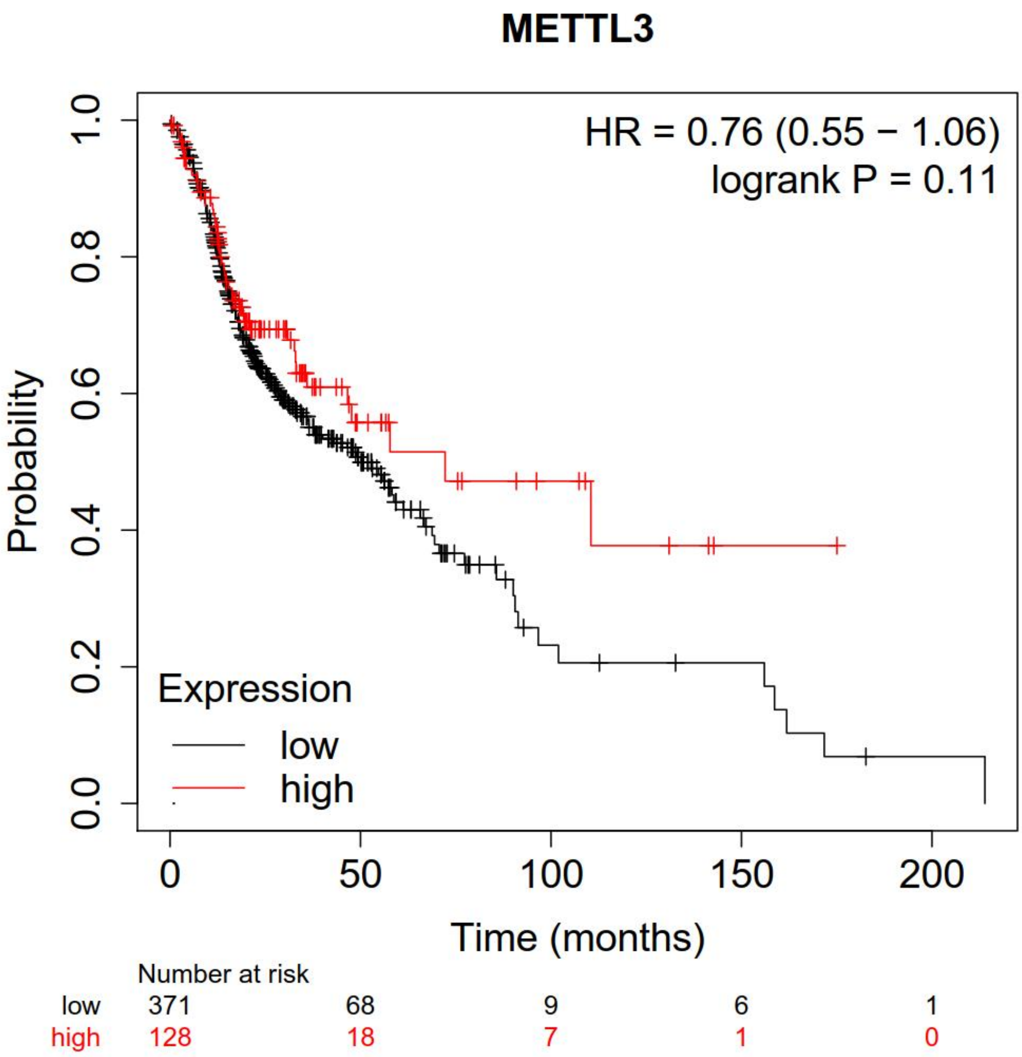

METTL14

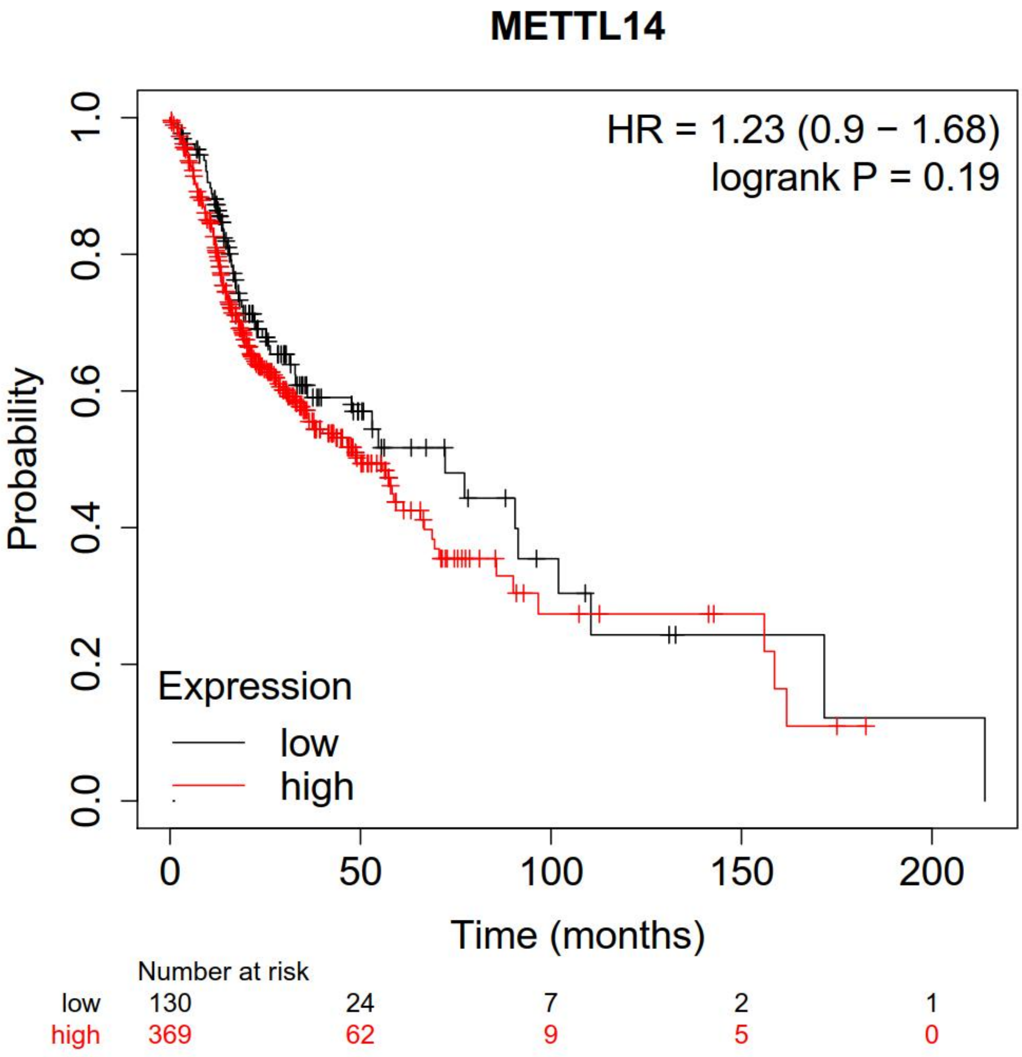
METTL14
HR = 1.23 (0.9 − 1.68)
logrank P = 0.19
Probability
1.0
0.8
0.6
0.4
0.2
0.0
Expression
low
high
0
50
100
150
200
Time (months)
Number at risk
low 130 24 7 2 1
high 369 62 9 5 0

METTL16

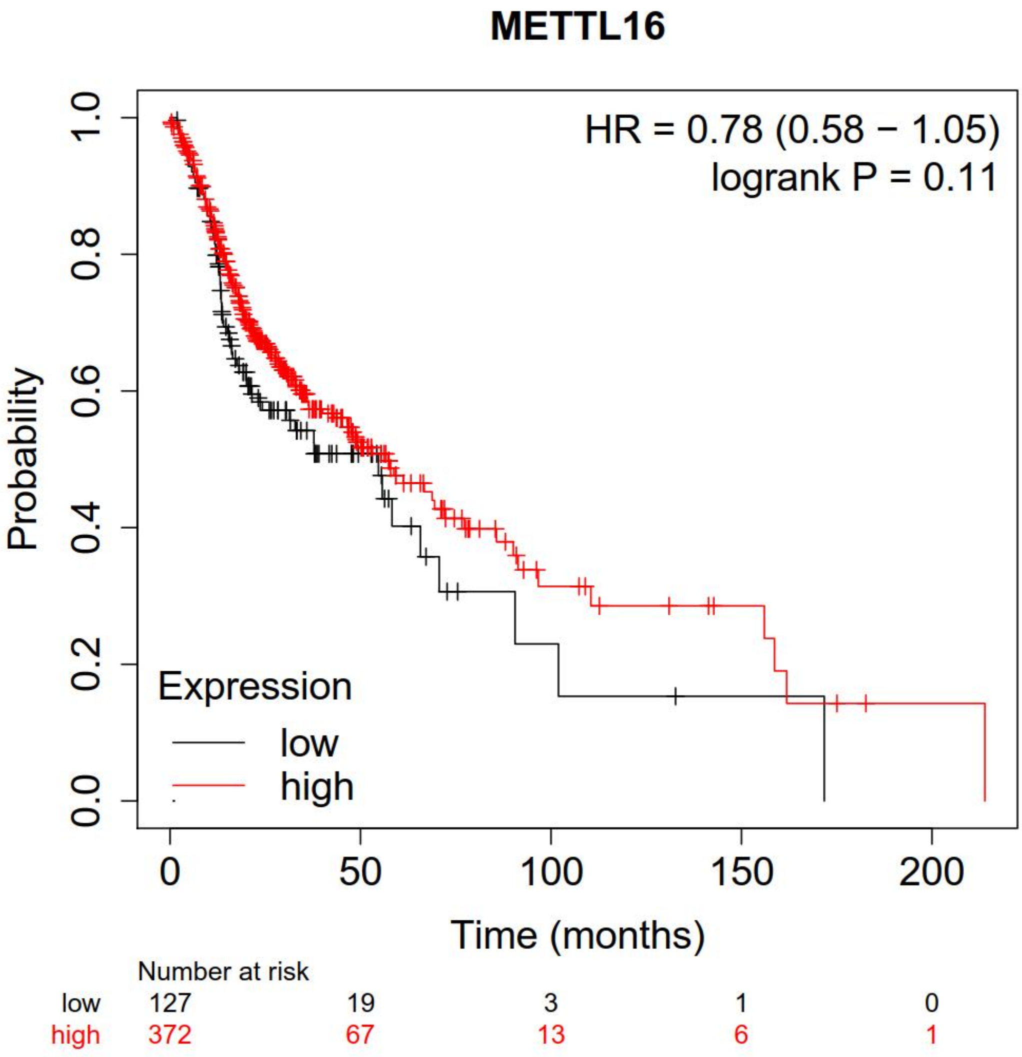
METTL16
HR = 0.78 (0.58 − 1.05)
logrank P = 0.11
Probability
1.0
0.8
0.6
0.4
0.2
0.0
Expression
low
high
0
50
100
150
200
Time (months)
Number at risk
low 127 19 3 1 0
high 372 67 13 6 1

WTAP

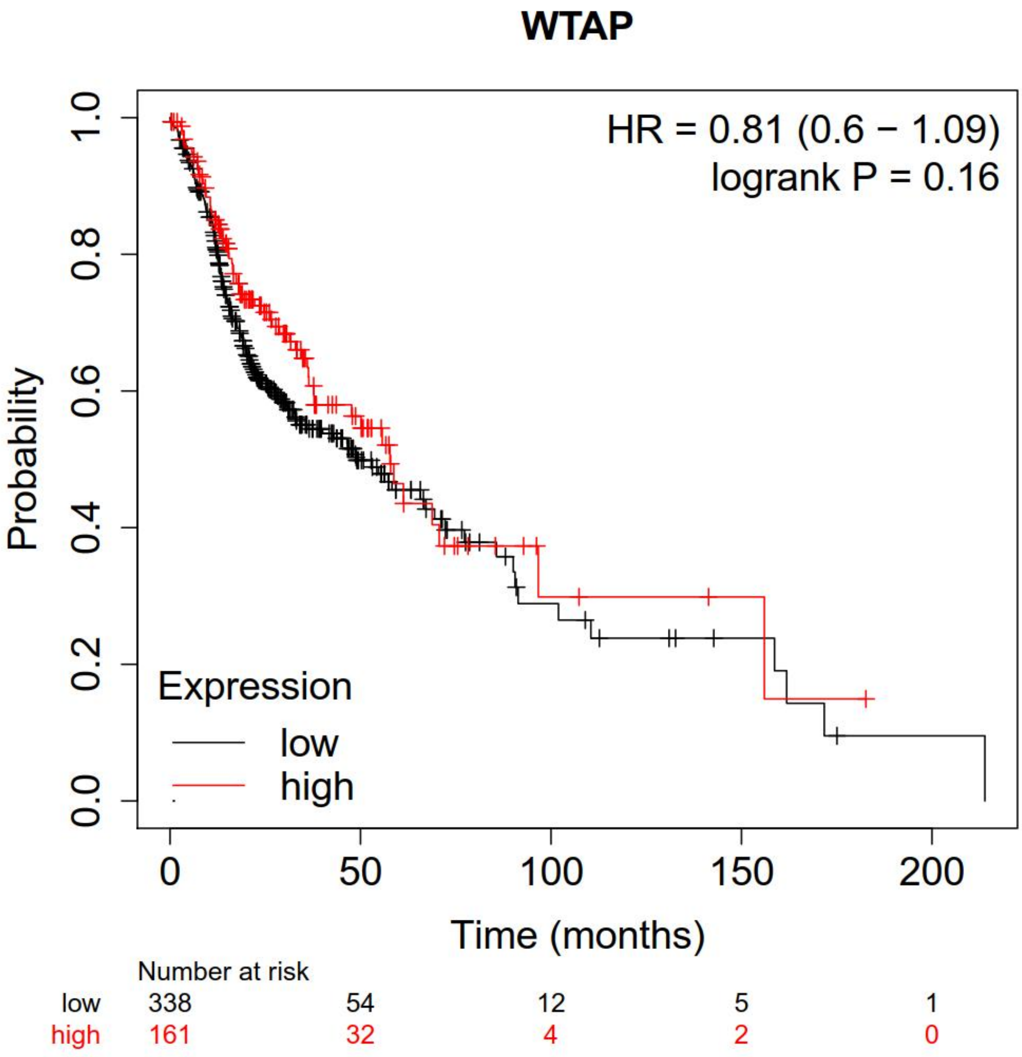
WTAP
HR = 0.81 (0.6 − 1.09)
logrank P = 0.16
Probability
0.0
0.2
0.4
0.6
0.8
1.0
Expression
low
high
0
50
100
150
200
Time (months)
Number at risk
low 338 54 12 5 1
high 161 32 4 2 0

RBM15B

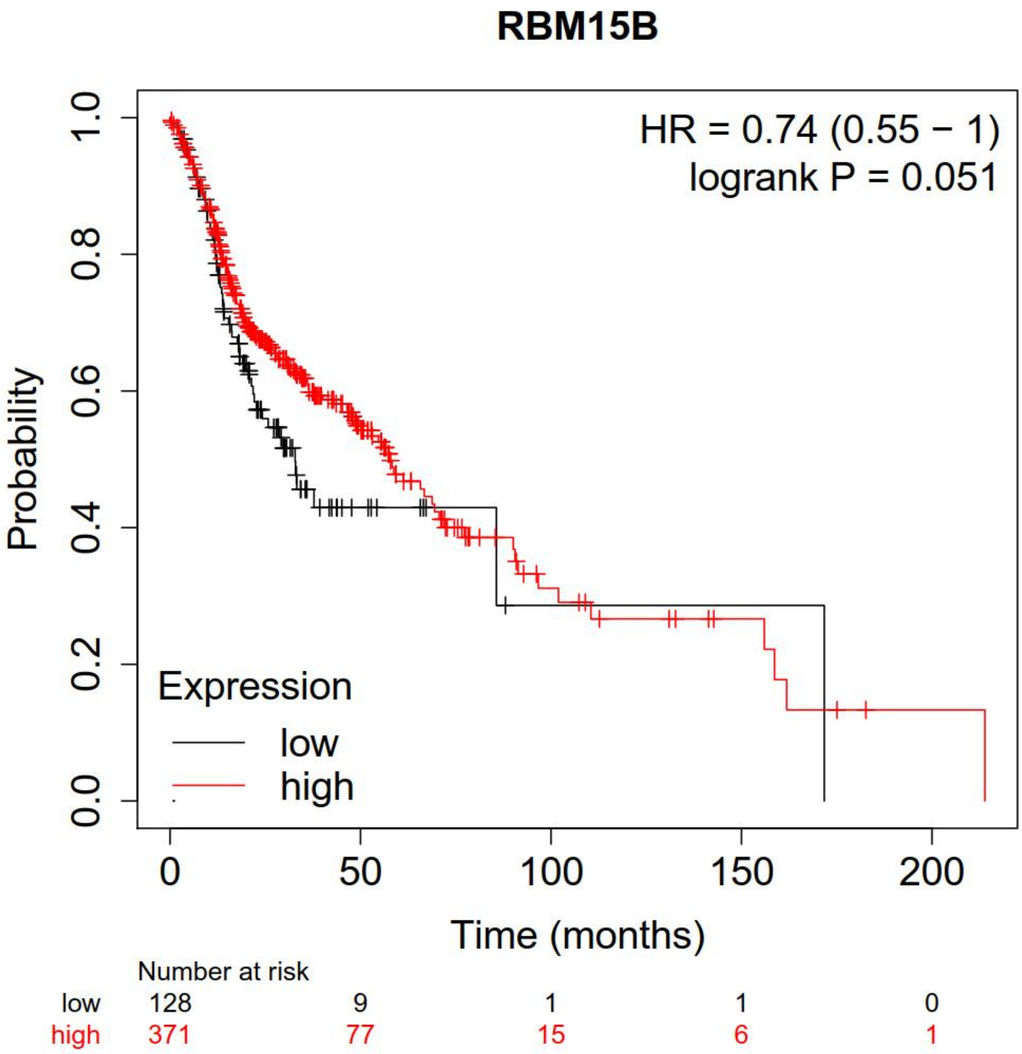

RBM15B
HR = 0.74 (0.55 − 1)
logrank P = 0.051
Probability
0.0
0.2
0.4
0.6
0.8
1.0
Expression
low
high
0
50
100
150
200
Time (months)
Number at risk
low 128 9 1 1 0
high 371 77 15 6 1

VIRMA

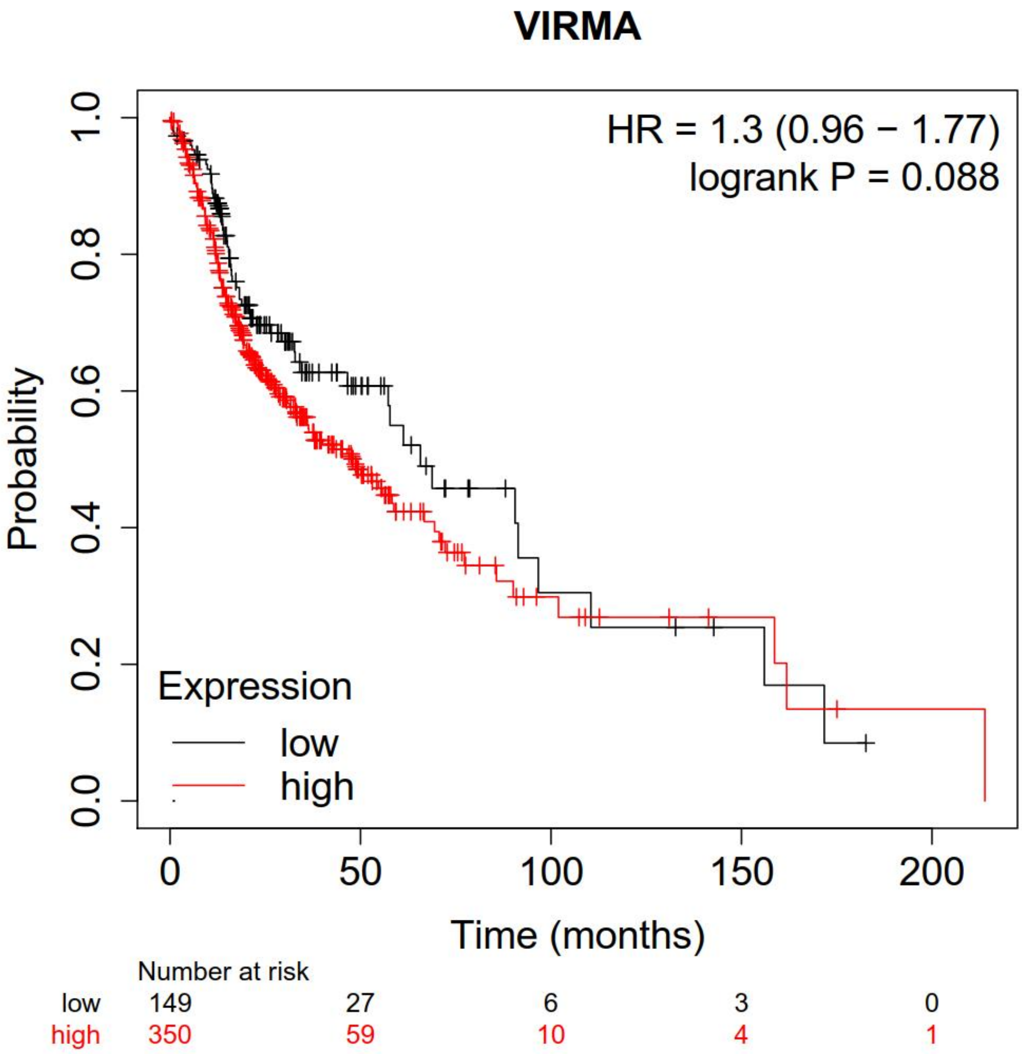

VIRMA
HR = 1.3 (0.96 − 1.77)
logrank P = 0.088
Probability
1.0
0.8
0.6
0.4
0.2
0.0
Expression
low
high
0
50
100
150
200
Time (months)
Number at risk
low 149 27 6 3 0
high 350 59 10 4 1

ZC3H13

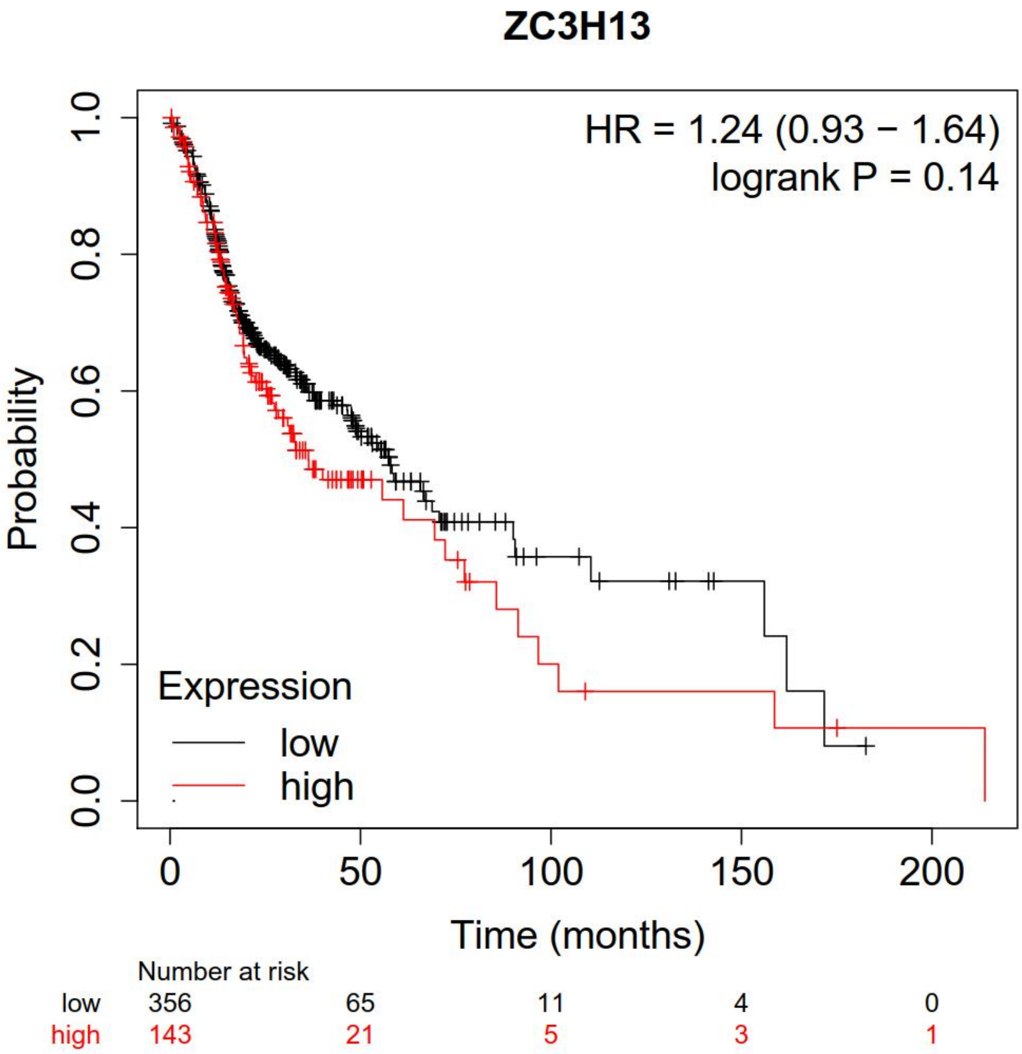

ZC3H13
HR = 1.24 (0.93 − 1.64)
logrank P = 0.14
Probability
0.0
0.2
0.4
0.6
0.8
1.0
Expression
low
high
0
50
100
150
200
Time (months)
Number at risk
low 356 65 11 4 0
high 143 21 5 3 1

RBM15

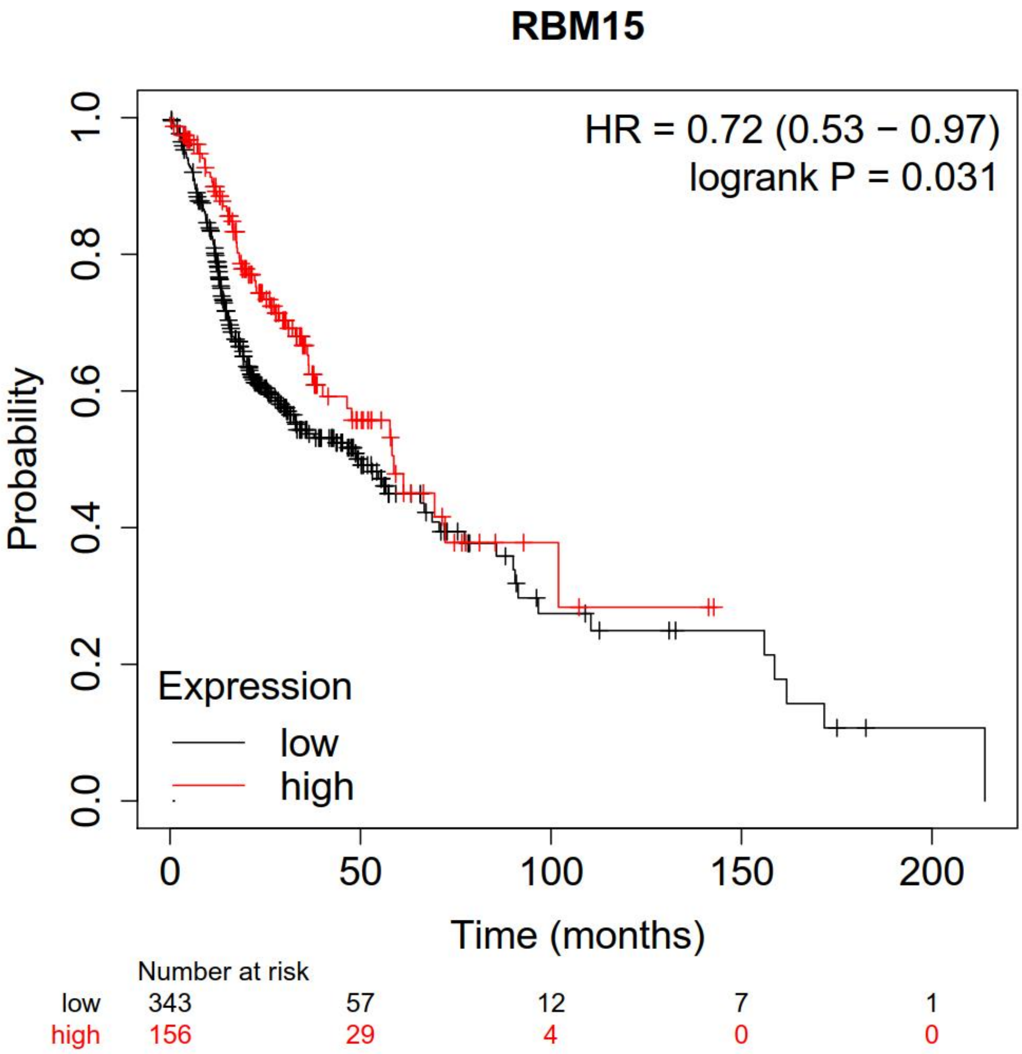
RBM15
HR = 0.72 (0.53 − 0.97)
logrank P = 0.031
Probability
0.0
0.2
0.4
0.6
0.8
1.0
Expression
low
high
0
50
100
150
200
Time (months)
Number at risk
low 343 57 12 7 1
high 156 29 4 0 0

ALKBH5

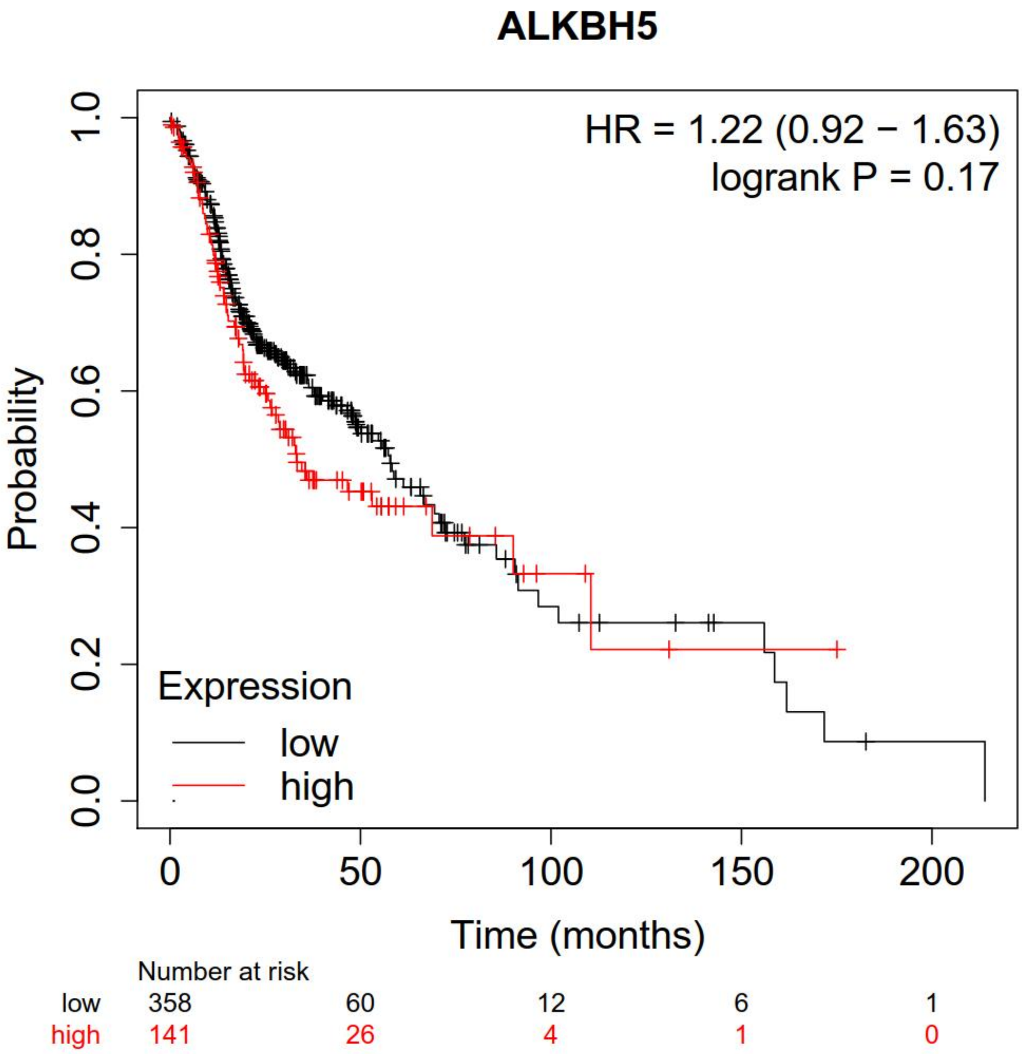

ALKBH5
HR = 1.22 (0.92 − 1.63)
logrank P = 0.17
Probability
1.0
0.8
0.6
0.4
0.2
0.0
Expression
low
high
0
50
100
150
200
Time (months)
Number at risk
low 358 60 12 6 1
high 141 26 4 1 0

FTO

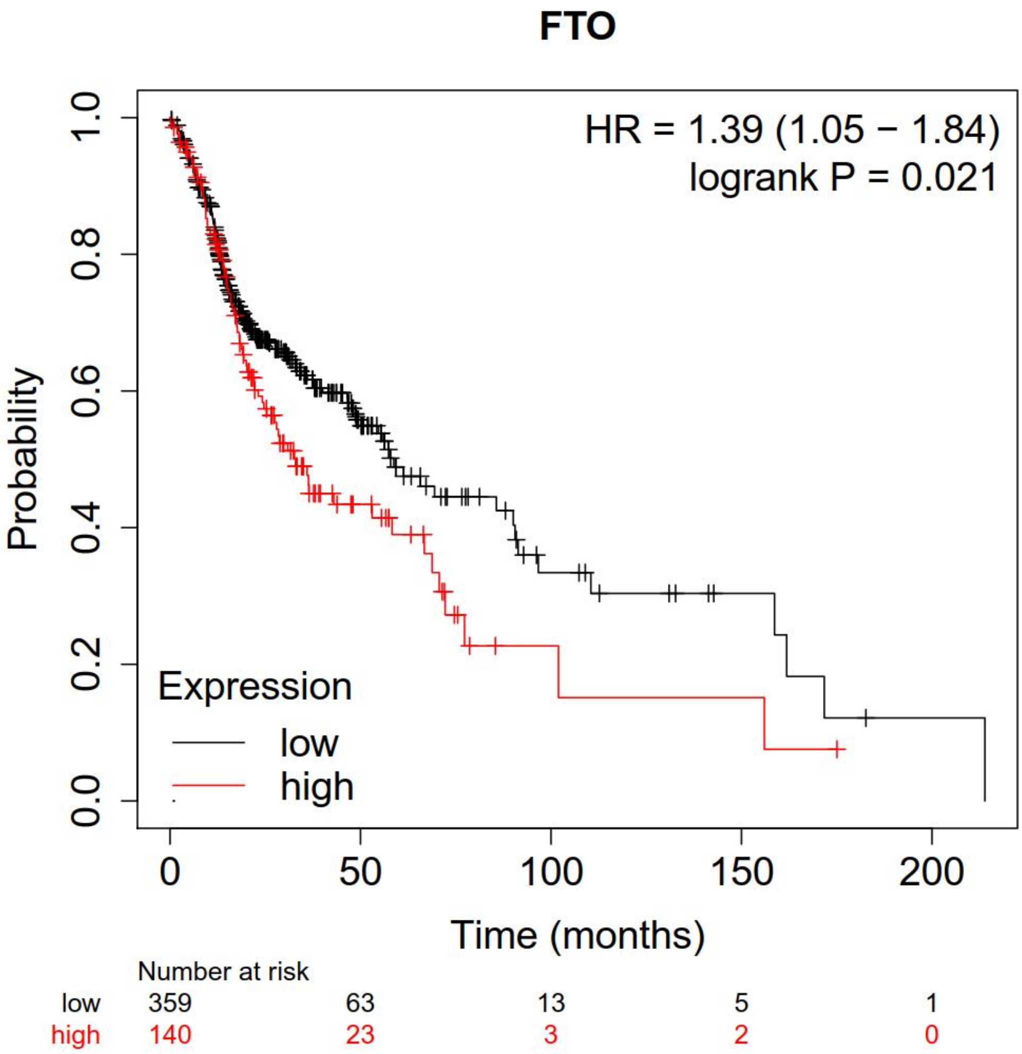
FTO
HR = 1.39 (1.05 − 1.84)
logrank P = 0.021
Probability
0.0
0.2
0.4
0.6
0.8
1.0
Expression
low
high
0
50
100
150
200
Time (months)
Number at risk
low 359 63 13 5 1
high 140 23 3 2 0

YTHDF1

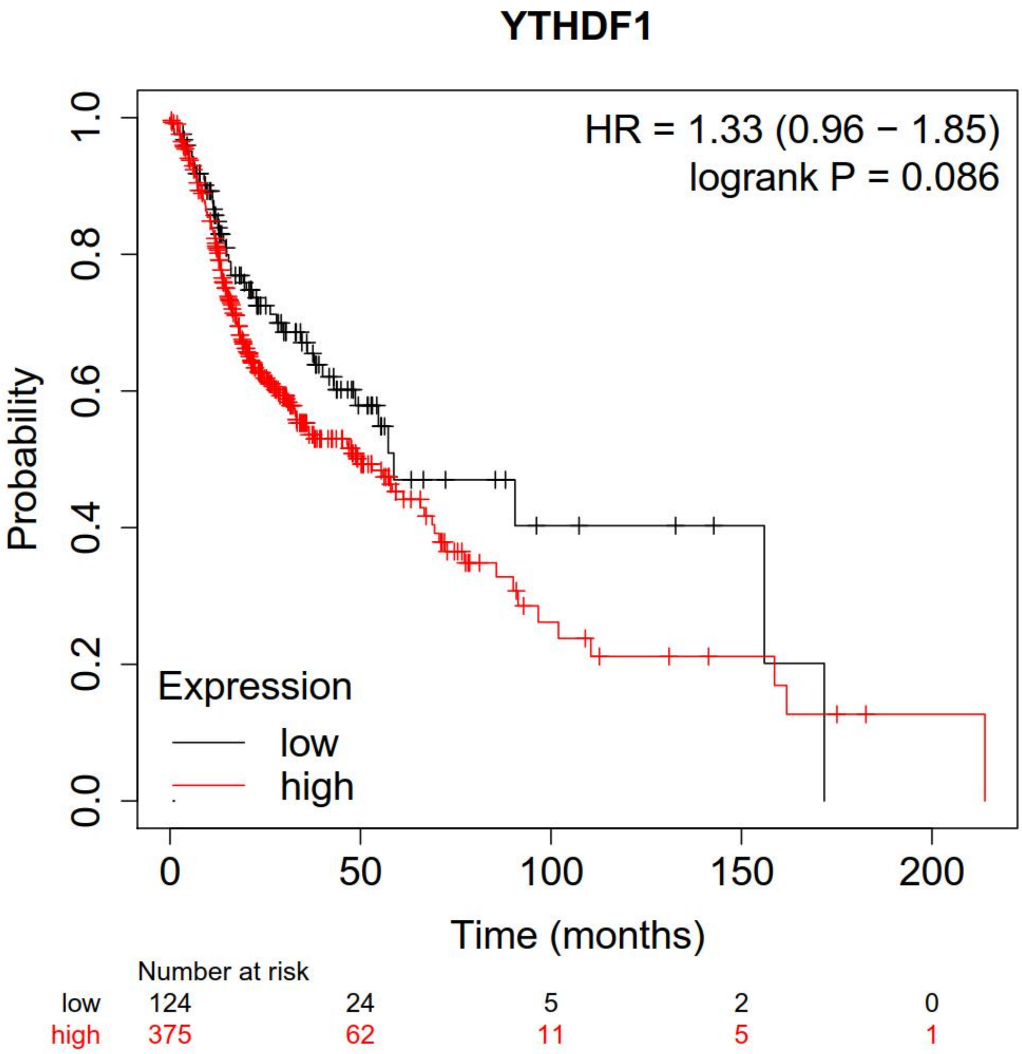
YTHDF1
HR = 1.33 (0.96 − 1.85)
logrank P = 0.086
Probability
1.0
0.8
0.6
0.4
0.2
0.0
Expression
low
high
0
50
100
150
200
Time (months)
Number at risk
low 124 24 5 2 0
high 375 62 11 5 1

YTHDF2

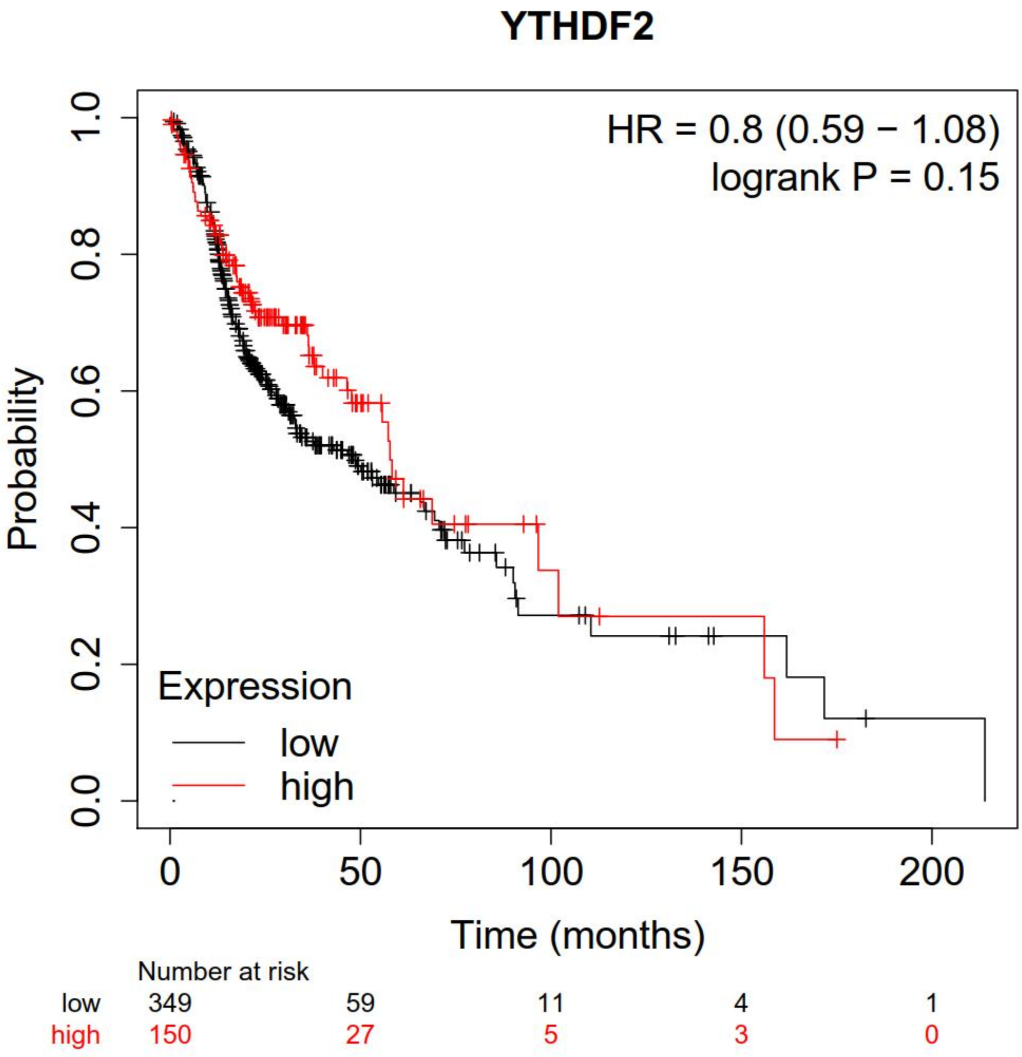
YTHDF2
HR = 0.8 (0.59 − 1.08)
logrank P = 0.15
Probability
0.0
0.2
0.4
0.6
0.8
1.0
Expression
low
high
0
50
100
150
200
Time (months)
Number at risk
low 349 59 11 4 1
high 150 27 5 3 0

YTHDF3

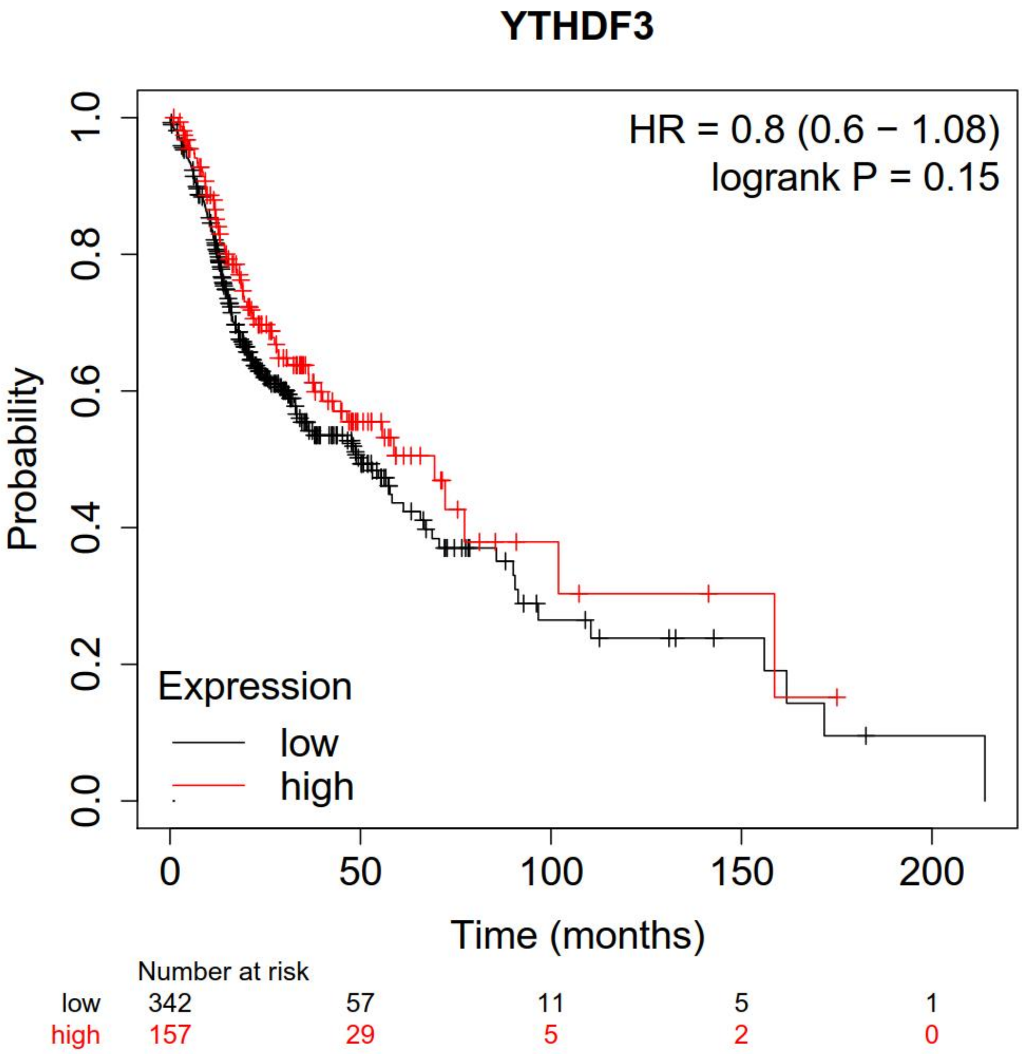
YTHDF3
HR = 0.8 (0.6 − 1.08)
logrank P = 0.15
Probability
0.0
0.2
0.4
0.6
0.8
1.0
Expression
low
high
0
50
100
150
200
Time (months)
Number at risk
low 342 57 11 5 1
high 157 29 5 2 0

RBMX

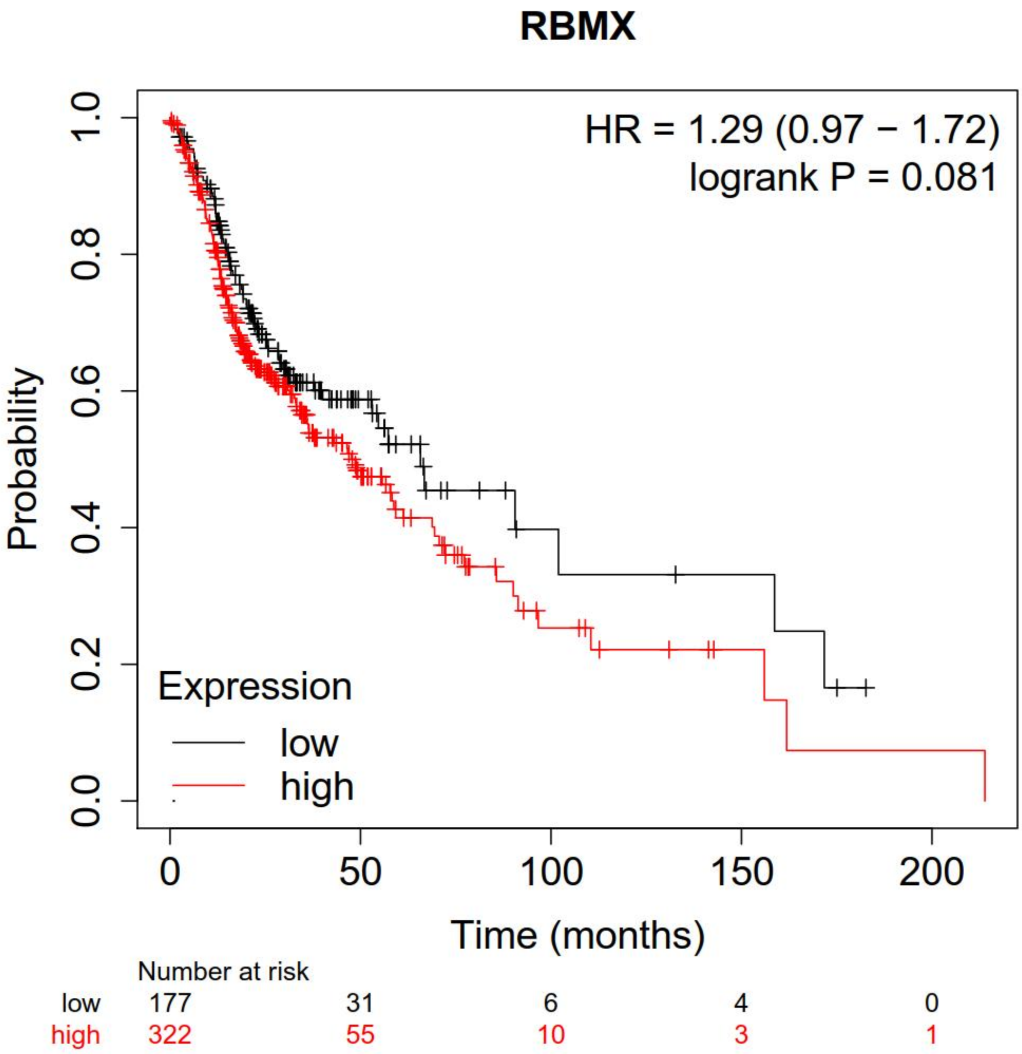
RBMX
HR = 1.29 (0.97 − 1.72)
logrank P = 0.081
Probability
0.0
0.2
0.4
0.6
0.8
1.0
Expression
low
high
0
50
100
150
200
Time (months)
Number at risk
low 177 31 6 4 0
high 322 55 10 3 1

FMR1

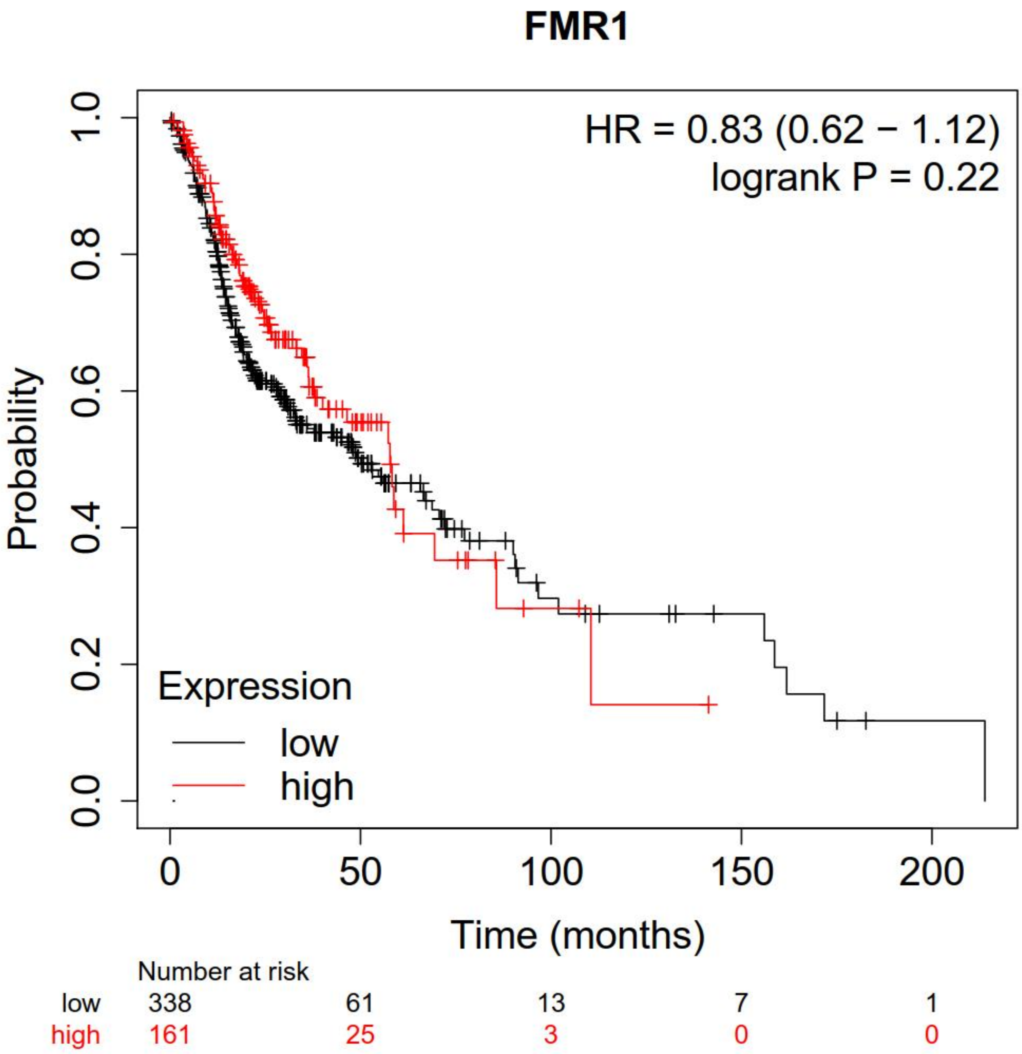

FMR1
HR = 0.83 (0.62 − 1.12)
logrank P = 0.22
Probability
0.0
0.2
0.4
0.6
0.8
1.0
Expression
low
high
0
50
100
150
200
Time (months)
Number at risk
low 338 61 13 7 1
high 161 25 3 0 0

HNRNPA2B1

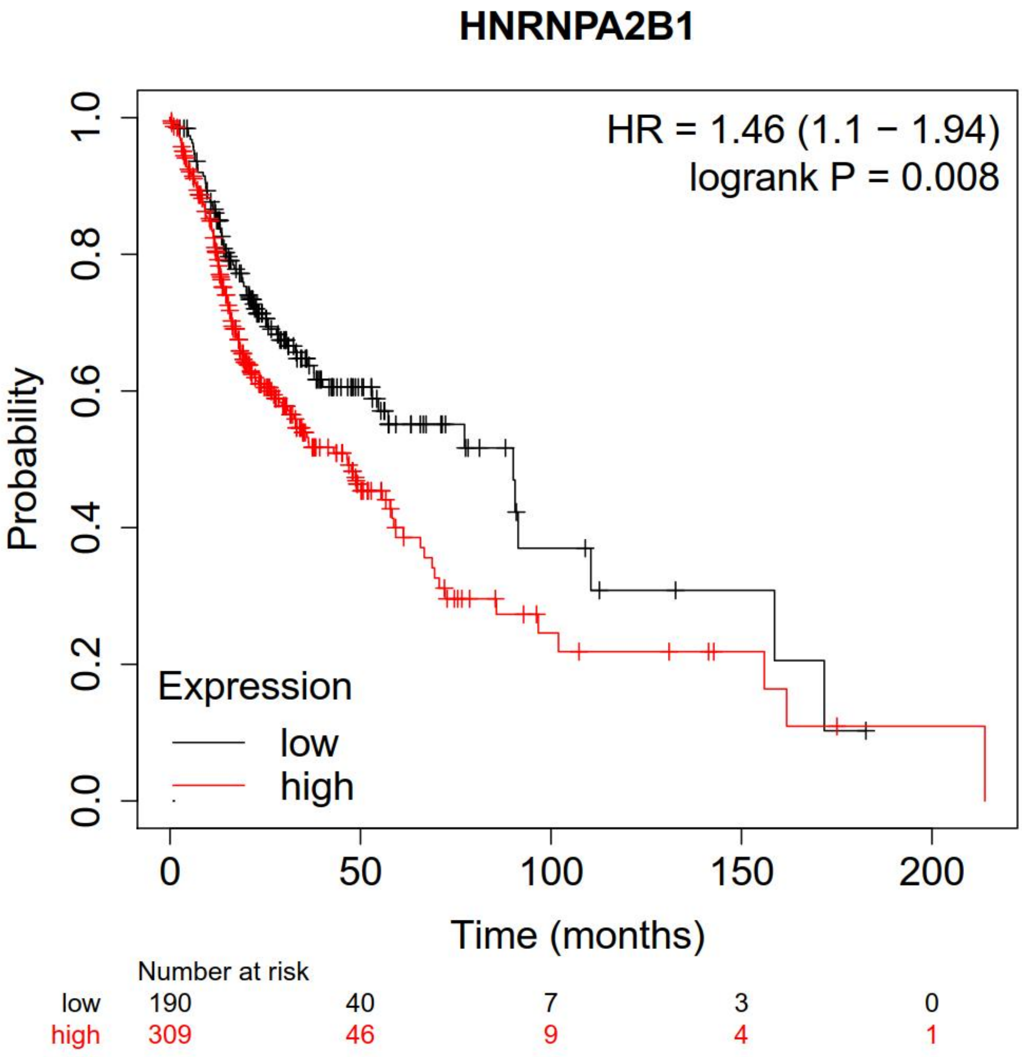
HNRNPA2B1
HR = 1.46 (1.1 − 1.94)
logrank P = 0.008
Probability
0.0
0.2
0.4
0.6
0.8
1.0
Expression
low
high
0
50
100
150
200
Time (months)
Number at risk
low 190 40 7 3 0
high 309 46 9 4 1

HNRNPC

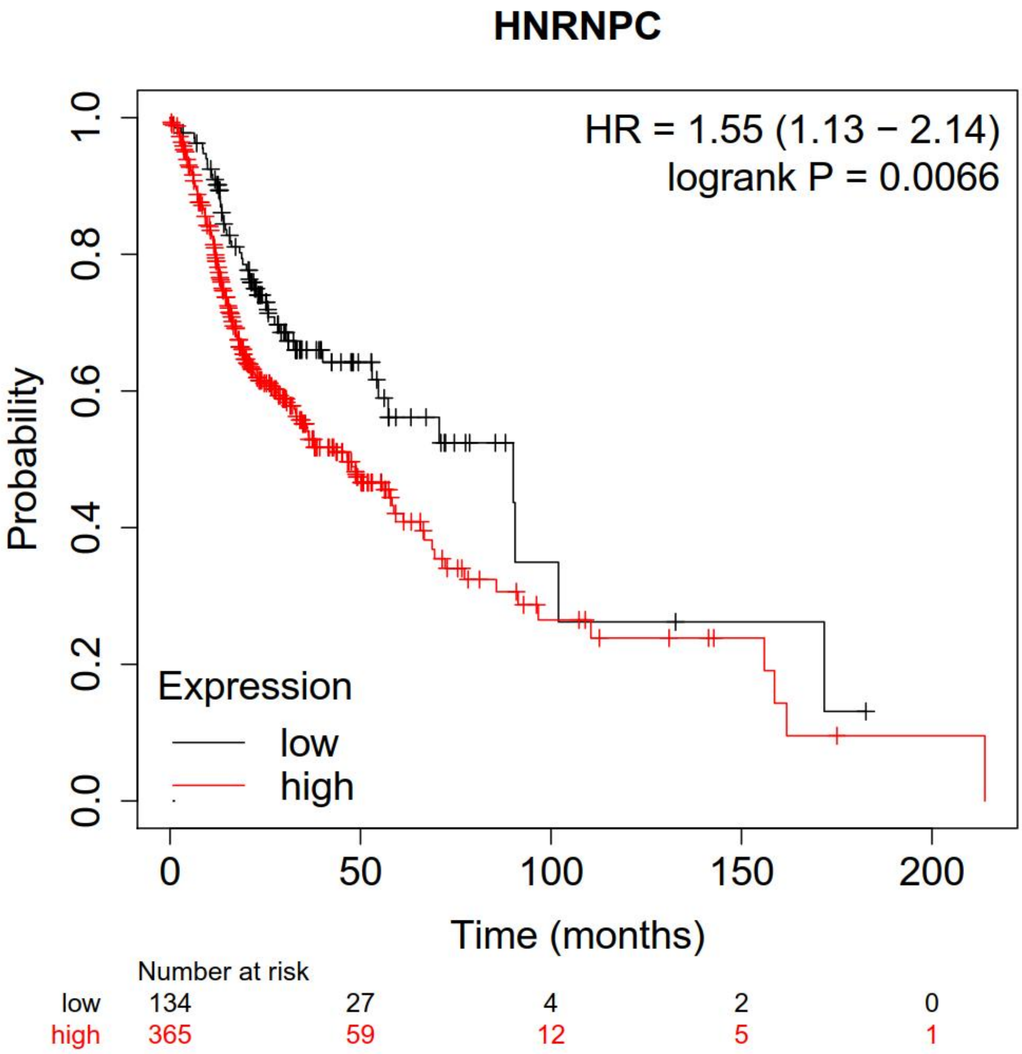
HNRNPC
HR = 1.55 (1.13 − 2.14)
logrank P = 0.0066
Probability
0.0
0.2
0.4
0.6
0.8
1.0
Expression
low
high
0
50
100
150
200
Time (months)
Number at risk
low 134 27 4 2 0
high 365 59 12 5 1

IGF2BP1

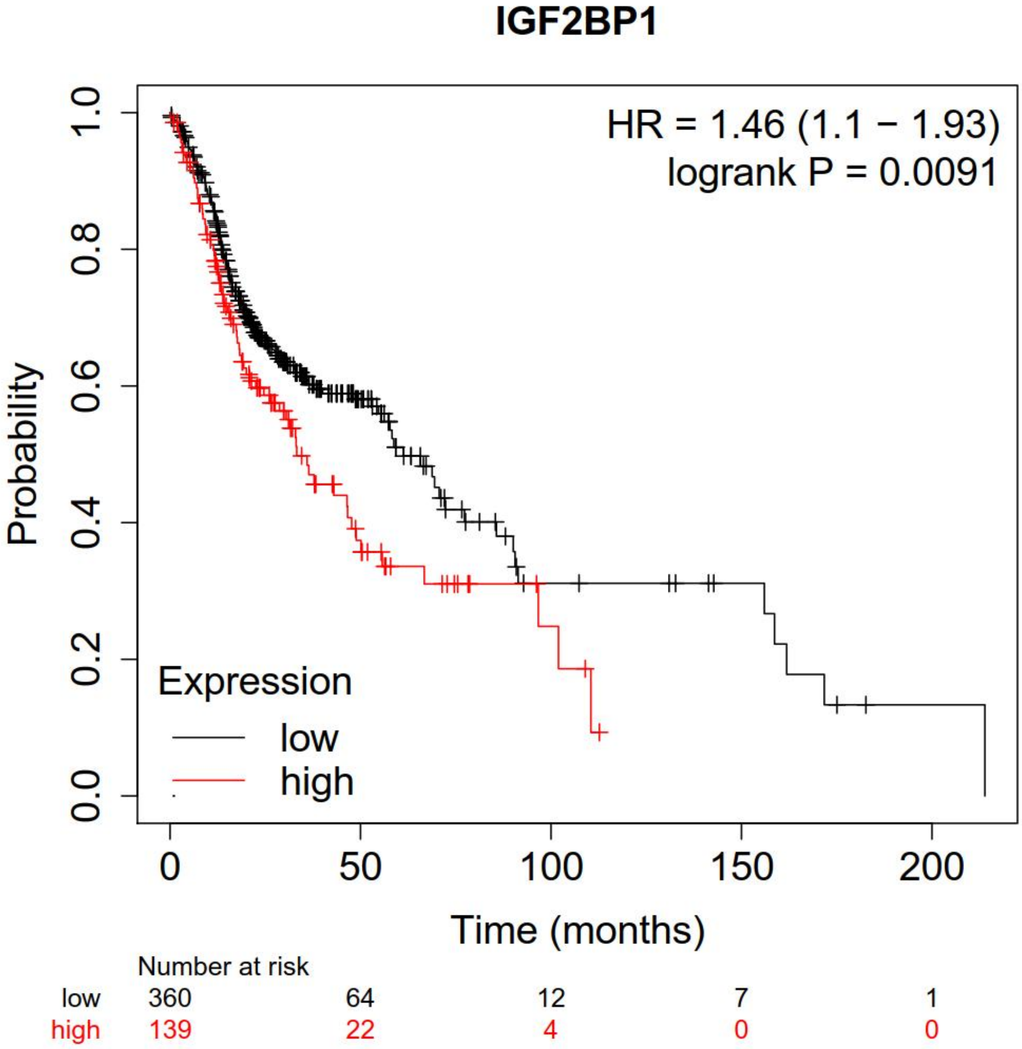
IGF2BP1
HR = 1.46 (1.1 − 1.93)
logrank P = 0.0091
Probability
0.0
0.2
0.4
0.6
0.8
1.0
Expression
low
high
0
50
100
150
200
Time (months)
Number at risk
low 360 64 12 7 1
high 139 22 4 0 0

YTHDC2

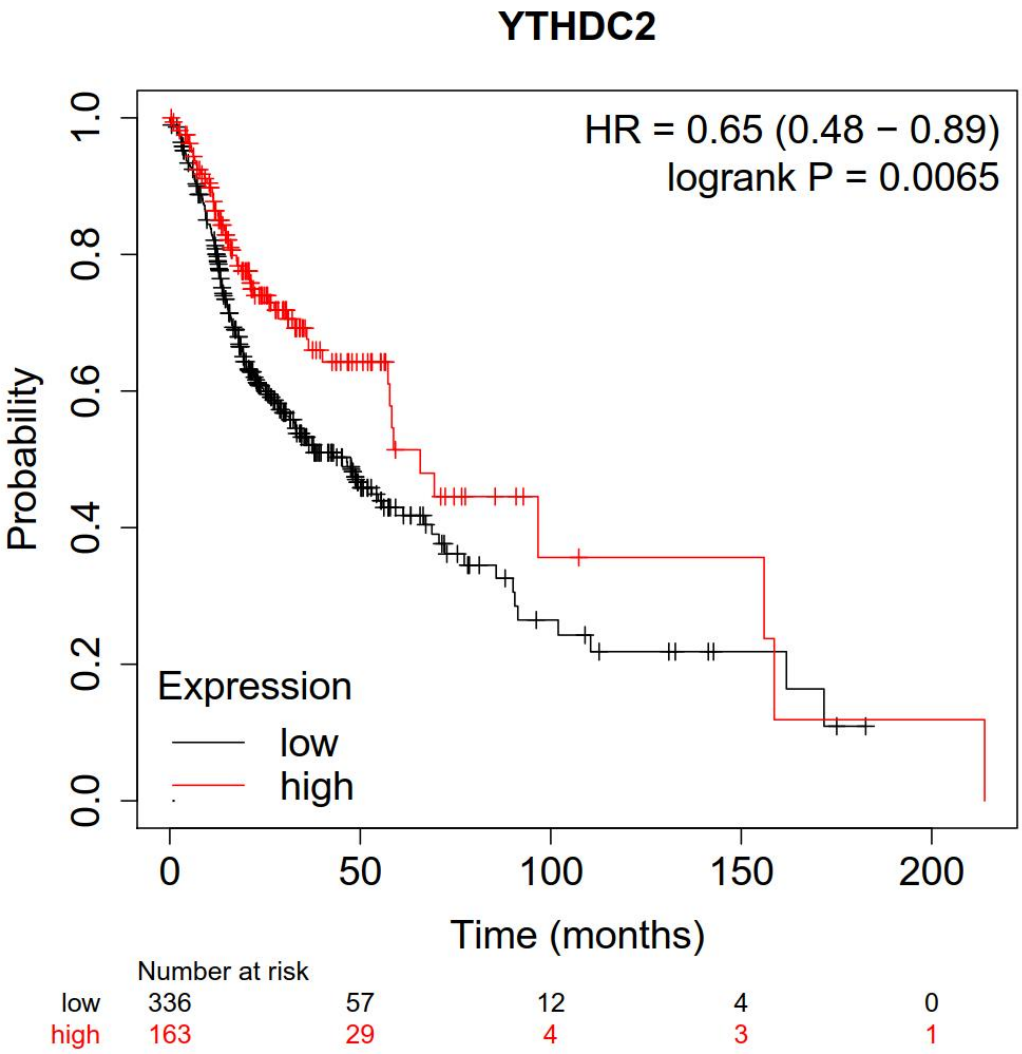
YTHDC2
HR = 0.65 (0.48 − 0.89)
logrank P = 0.0065
Probability
1.0
0.8
0.6
0.4
0.2
0.0
Expression
low
high
0
50
100
150
200
Time (months)
Number at risk
low 336 57 12 4 0
high 163 29 4 3 1

LRPPRC

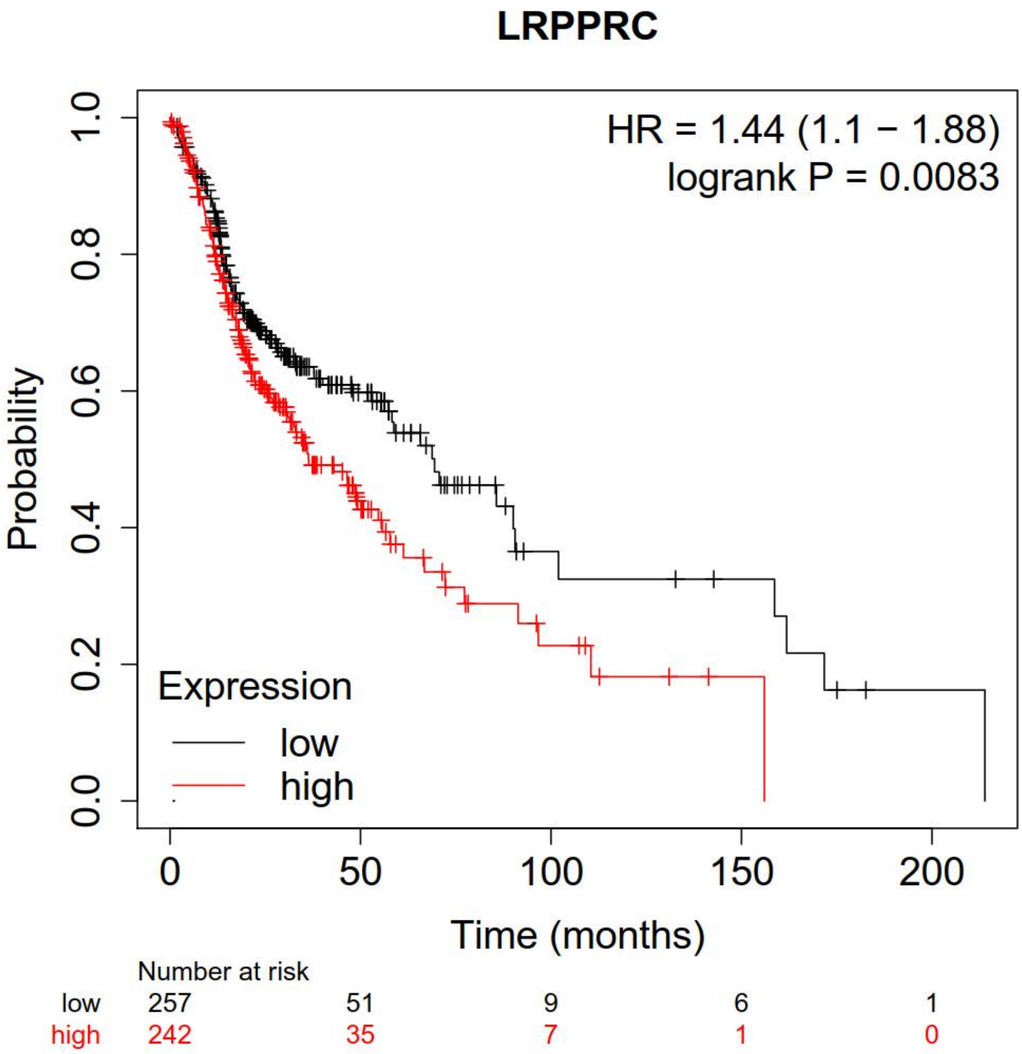
LRPPRC
HR = 1.44 (1.1 − 1.88)
logrank P = 0.0083
Probability
0.0
0.2
0.4
0.6
0.8
1.0
Expression
low
high
0
50
100
150
200
Time (months)
Number at risk
low 257 51 9 6 1
high 242 35 7 1 0

YTHDC1

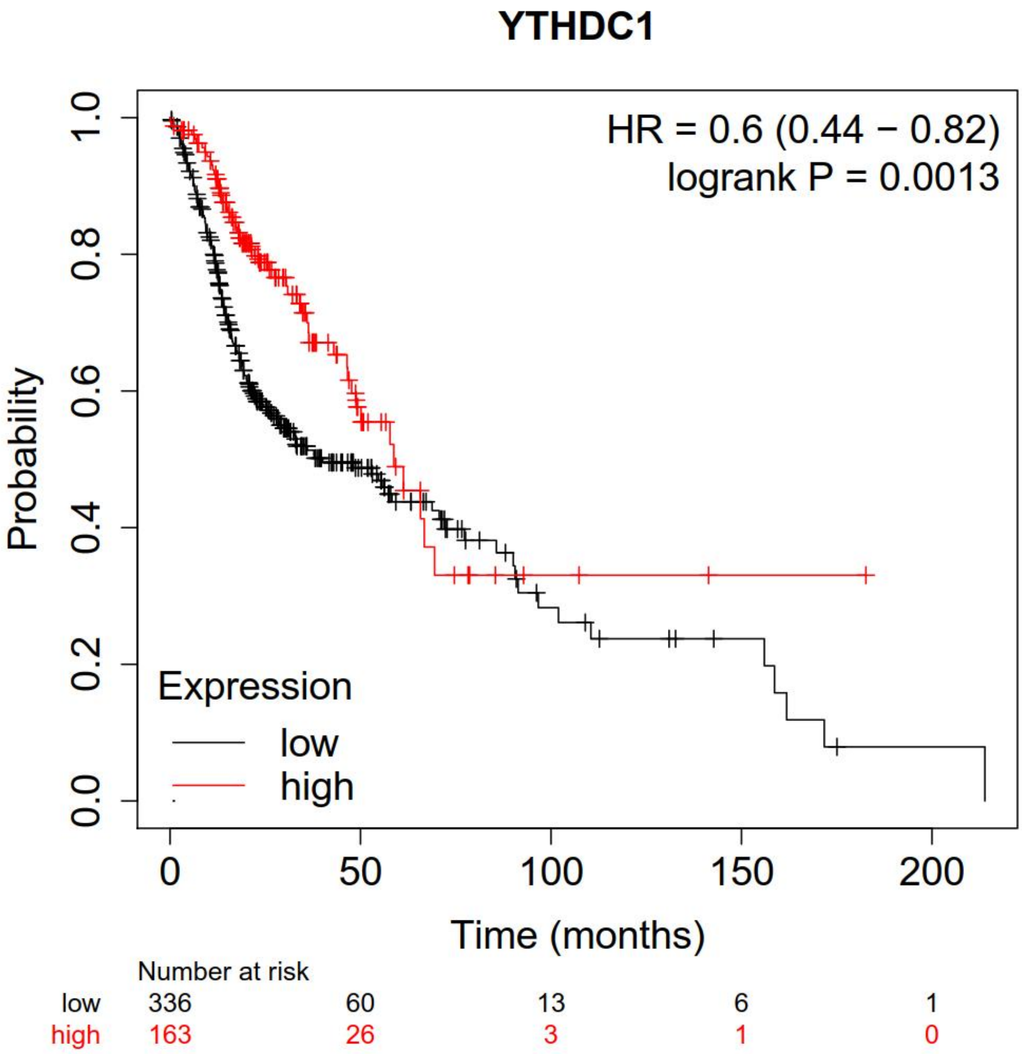
YTHDC1
HR = 0.6 (0.44 − 0.82)
logrank P = 0.0013
Probability
0.0
0.2
0.4
0.6
0.8
1.0
Expression
low
high
0
50
100
150
200
Time (months)
Number at risk
low 336 60 13 6 1
high 163 26 3 1 0

IGF2BP2

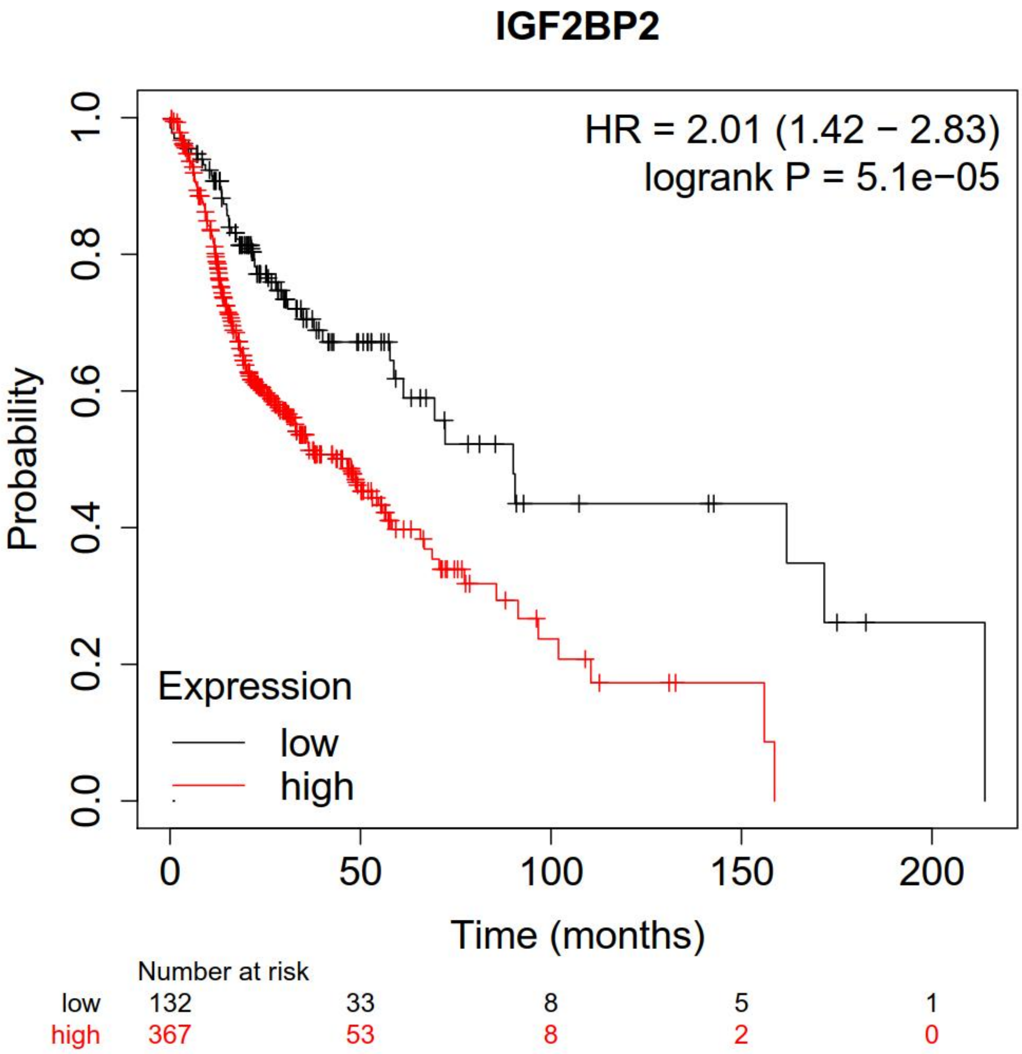

IGF2BP2
HR = 2.01 (1.42 − 2.83)
logrank P = 5.1e−05
Probability
1.0
0.8
0.6
0.4
0.2
0.0
Expression
low
high
0
50
100
150
200
Time (months)
Number at risk
low 132 33 8 5 1
high 367 53 8 2 0

IGF2BP3

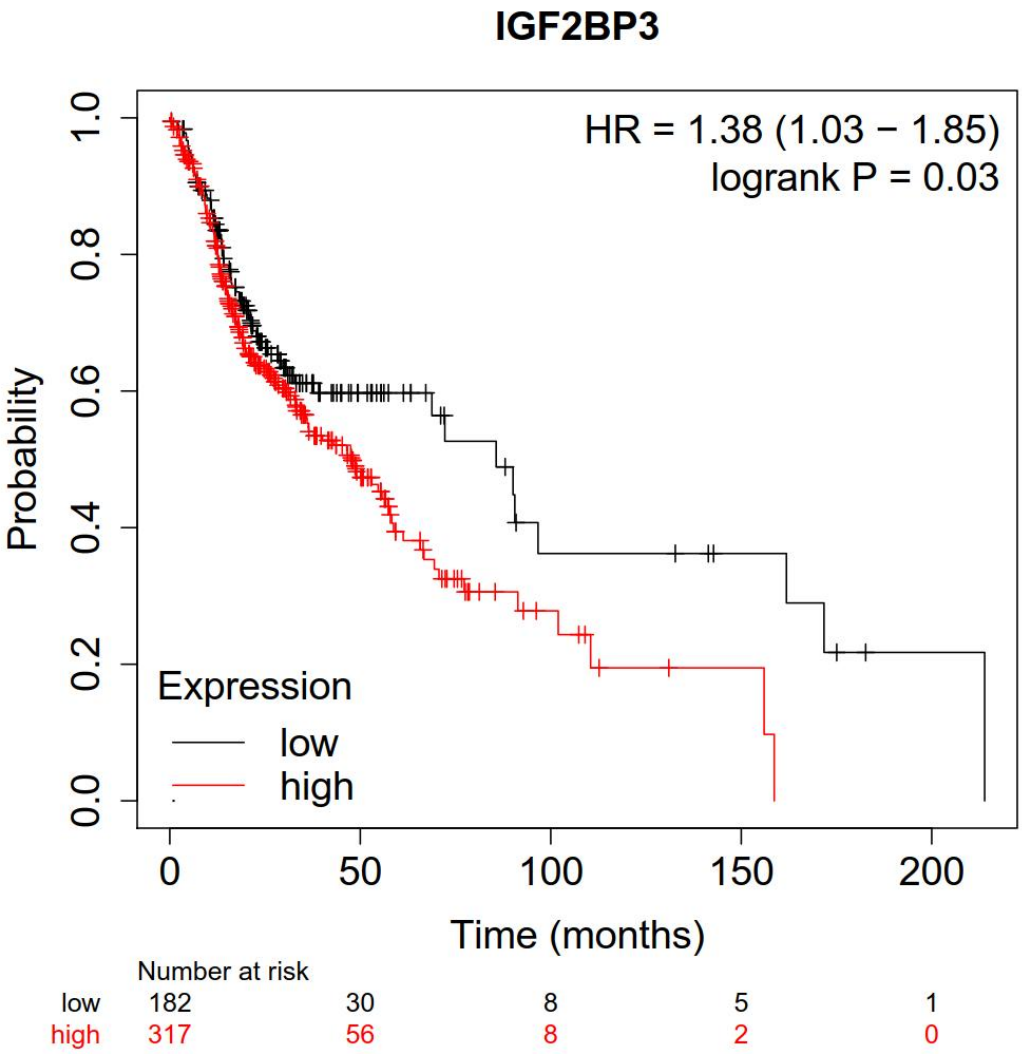

IGF2BP3
HR = 1.38 (1.03 − 1.85)
logrank P = 0.03
Probability
0.0
0.2
0.4
0.6
0.8
1.0
Expression
low
high
0
50
100
150
200
Time (months)
Number at risk
low 182 30 8 5 1
high 317 56 8 2 0
