# Supplementary figures and images for: Expression and significance of m6A-RNA-methylation in oral cancer and precancerous lesion
Source: Front Oncol. 2023 Jan 30;13:1013054. doi: 10.3389/fonc.2023.1013054 (PMC9923020; doi:10.3389/fonc.2023.1013054)

## GEPIA database

### IGF2BP2

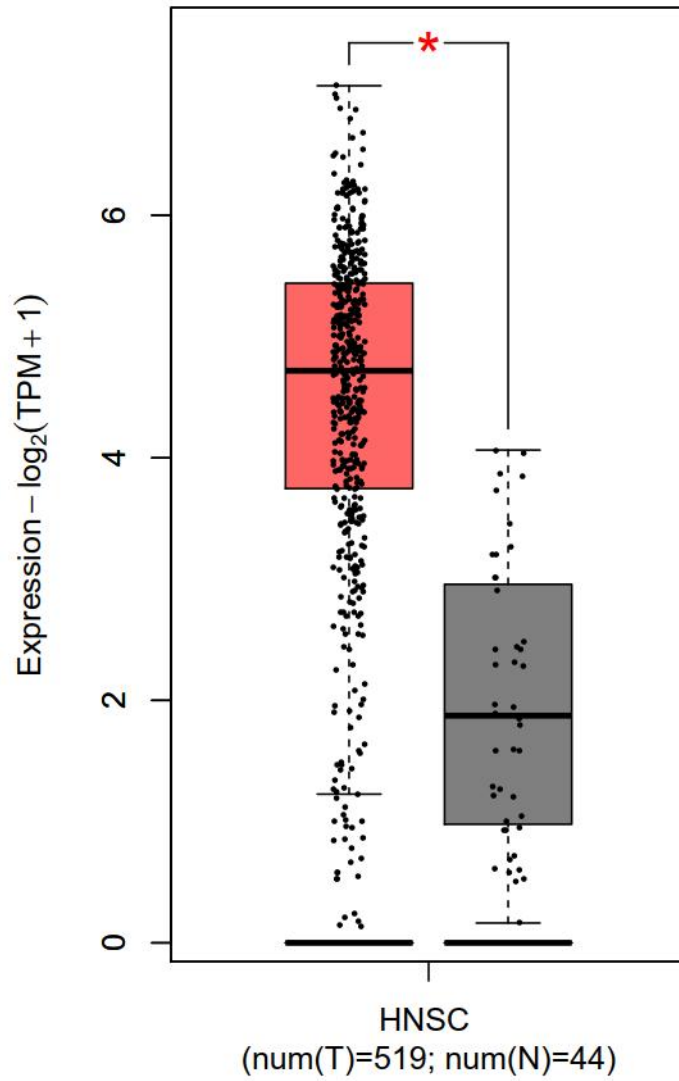

## IGF2BP3

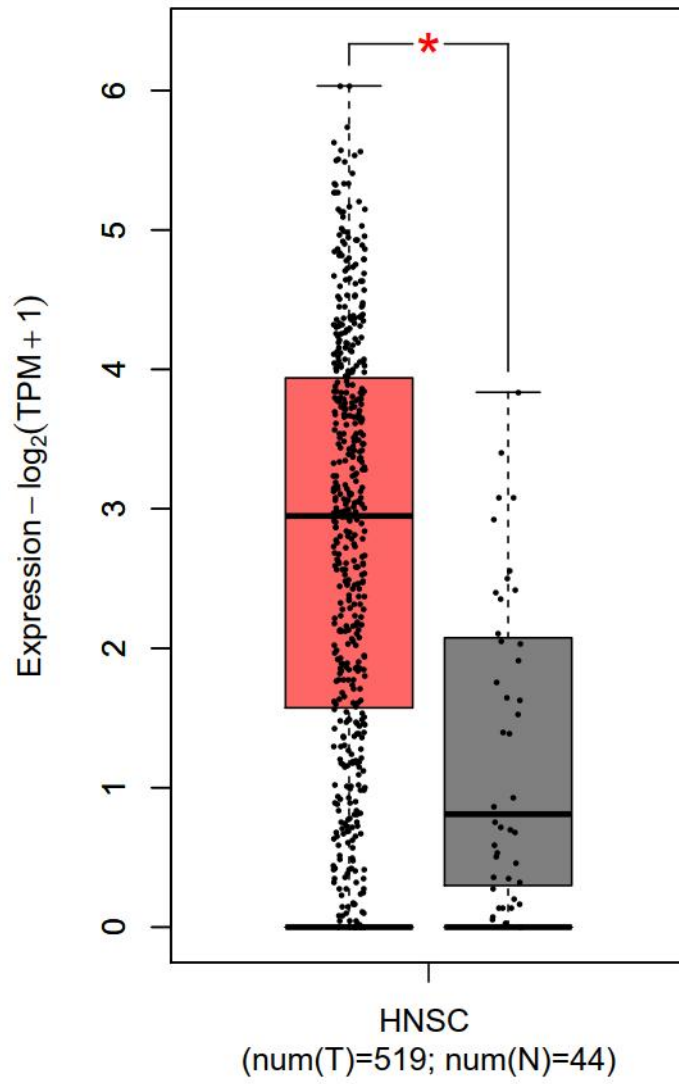

Supplement: Supplementary file 3 [file DataSheet_3.pdf]

cBioPortal database

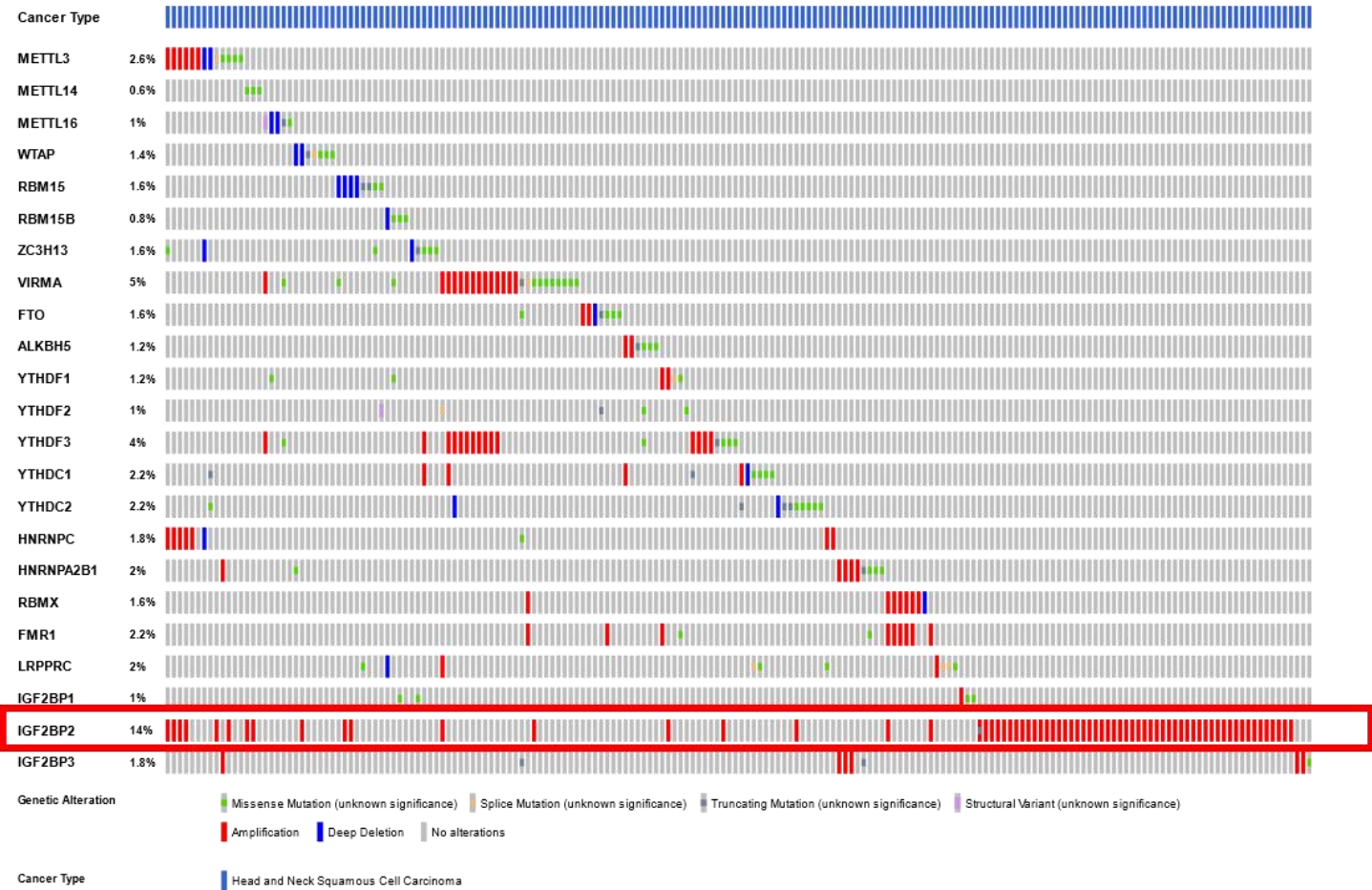

Supplement: Supplementary file 4 [file DataSheet_4.pdf]

TIMER database

IGF2BP2

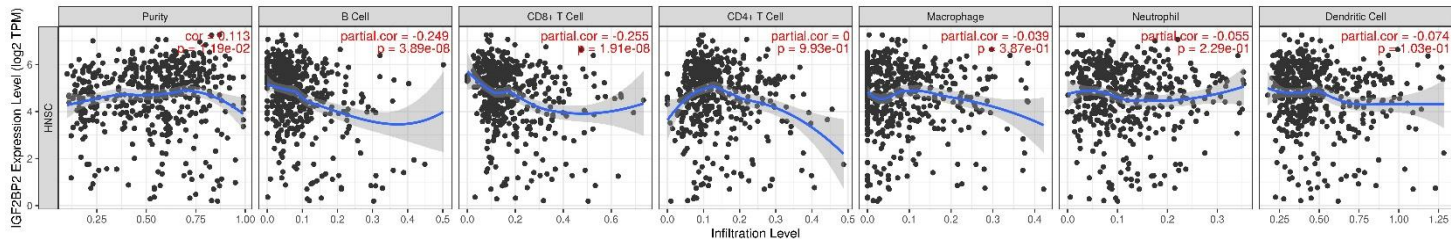

IGF2BP3

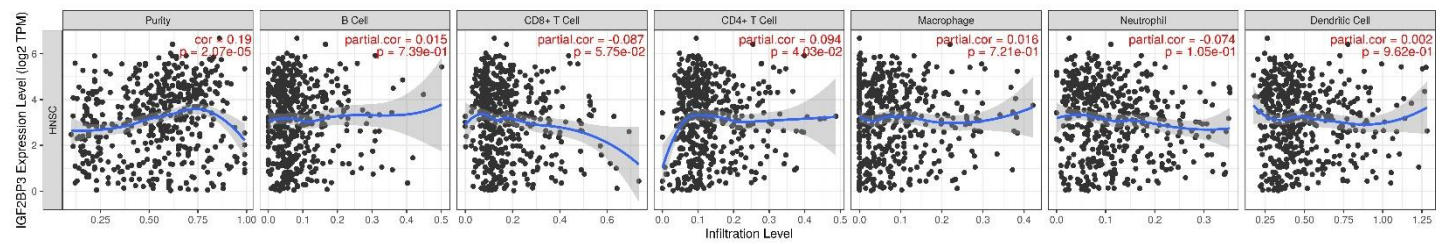

Supplement: Supplementary file 5 [file DataSheet_5.pdf]
